# Conductive single-wall carbon nanotubes/extracellular matrix hybrid hydrogels promote the lineage-specific development of seeding cells for tissue repair through reconstructing an integrin-dependent niche

**DOI:** 10.1186/s12951-021-00993-3

**Published:** 2021-08-23

**Authors:** Rui Bai, Jianfeng Liu, Jiao Zhang, Jinmiao Shi, Zhigeng Jin, Yi Li, Xiaoyu Ding, Xiaoming Zhu, Chao Yuan, Bingshui Xiu, Huiliang Liu, Zengqiang Yuan, Zhiqiang Liu

**Affiliations:** 1 Senior Department of Cardiology, The Sixth Medical Center of PLA General Hospital, Beijing, 100048 China; 2 Beijing Institute of Basic Medical Sciences, Beijing, 100850 China; 3 Department of Cardiology, The Second Medical Center & National Clinical Research Center for Geriatric Diseases, Chinese PLA General Hospital, Beijing, 100853 China; 4 Department of Cardiology, Beijing Electric Power Hospital, State Grid Corporation of China, Beijing, 100073 China

**Keywords:** Hybrid hydrogel, Single-wall carbon nanotubes, Extracellular matrixes, Regenerative medicine, Bioactive scaffolds

## Abstract

**Background:**

The niche of tissue development in vivo involves the growth matrix, biophysical cues and cell-cell interactions. Although natural extracellular matrixes may provide good supporting for seeding cells in vitro, it is evitable to destroy biophysical cues during decellularization. Reconstructing the bioactivities of extracellular matrix-based scaffolds is essential for their usage in tissue repair.

**Results:**

In the study, a hybrid hydrogel was developed by incorporating single-wall carbon nanotubes (SWCNTs) into heart-derived extracellular matrixes. Interestingly, insoluble SWCNTs were well dispersed in hybrid hydrogel solution via the interaction with extracellular matrix proteins. Importantly, an augmented integrin-dependent niche was reconstructed in the hybrid hydrogel, which could work like biophysical cues to activate integrin-related pathway of seeding cells. As supporting scaffolds in vitro, the hybrid hydrogels were observed to significantly promote seeding cell adhesion, differentiation, as well as structural and functional development towards mature cardiac tissues. As injectable carrier scaffolds in vivo, the hybrid hydrogels were then used to delivery stem cells for myocardial repair in rats. Similarly, significantly enhanced cardiac differentiation and maturation(12.5 ± 2.3% VS 32.8 ± 5%) of stem cells were detected in vivo, resulting in improved myocardial regeneration and repair.

**Conclusions:**

The study represented a simple and powerful approach for exploring bioactive scaffold to promote stem cell-based tissue repair.

**Graphic abstract:**

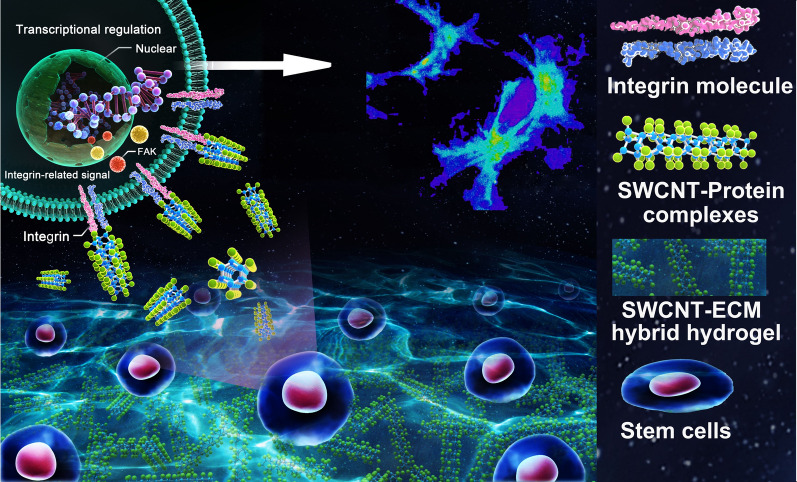

**Supplementary Information:**

The online version contains supplementary material available at 10.1186/s12951-021-00993-3.

## Introduction

Regenerative medicine employed biomaterials, seeding cells and cytokines to repair injured tissues, or construct artificial tissues for tissue even organ replacement [1–3]. The regenerative strategy was especially important for those tissues with limited regenerative potentials, such as adult myocardium and nerve [4, 5]. Therefore, myocardial repair or regeneration was one of the most concerned areas in regenerative medicine [6, 7].

Scaffold is one of the key factors in regenerative medicine. They may play multiple roles in tissue repair, such as providing temporary matrix for cell growth, promoting seeding cell survival and engraftment, regulating cell fate, and so on [8–11]. In the past years, various scaffolds were developed for myocardial repair, including natural and synthetic ones [12, 13]. Independent groups have focused on improving the bioactivity as well as other properties of scaffolds, aiming to make them mimic to the natural extracellular matrix (ECM) as much as possible [14–16]. For the purpose, decellularized technique of natural organs was developed which brought about ECM-based materials [17–19]. Through decellularization, cells were removed and ECM components were largely preserved. In addition, well-decellularized tissues may also preserve complete vascular structures, allowing for the ingrowth of new vessel during tissue construction. Because of their natural organ source, ECM materials should be the optimal ones among various scaffolds to provide the closest conditions to in vivo microenvironments for seeding cells [20]. Due to the development of ECM-based materials, several breakthroughs in regenerative medicine have been achieved. A representative one was the successful construction of an artificial heart with rhythmic beating using the whole heart decellularized matrix and primary cardiomyocytes [21, 22]. Besides the solid scaffolds, ECM-based hydrogels were also developed which could be used as injectable carriers, more convenient for cell delivery [17, 23]. Despite the outstanding performance of ECM-based materials, the decellularization process is evitable to impair niches in natural tissues, leading to the loss of some bioactivities. Reconstructing a functional niche in decellularized ECM would be significant to improve such scaffolds in guiding tissue-specific regeneration [24, 25].

**Scheme 1:**
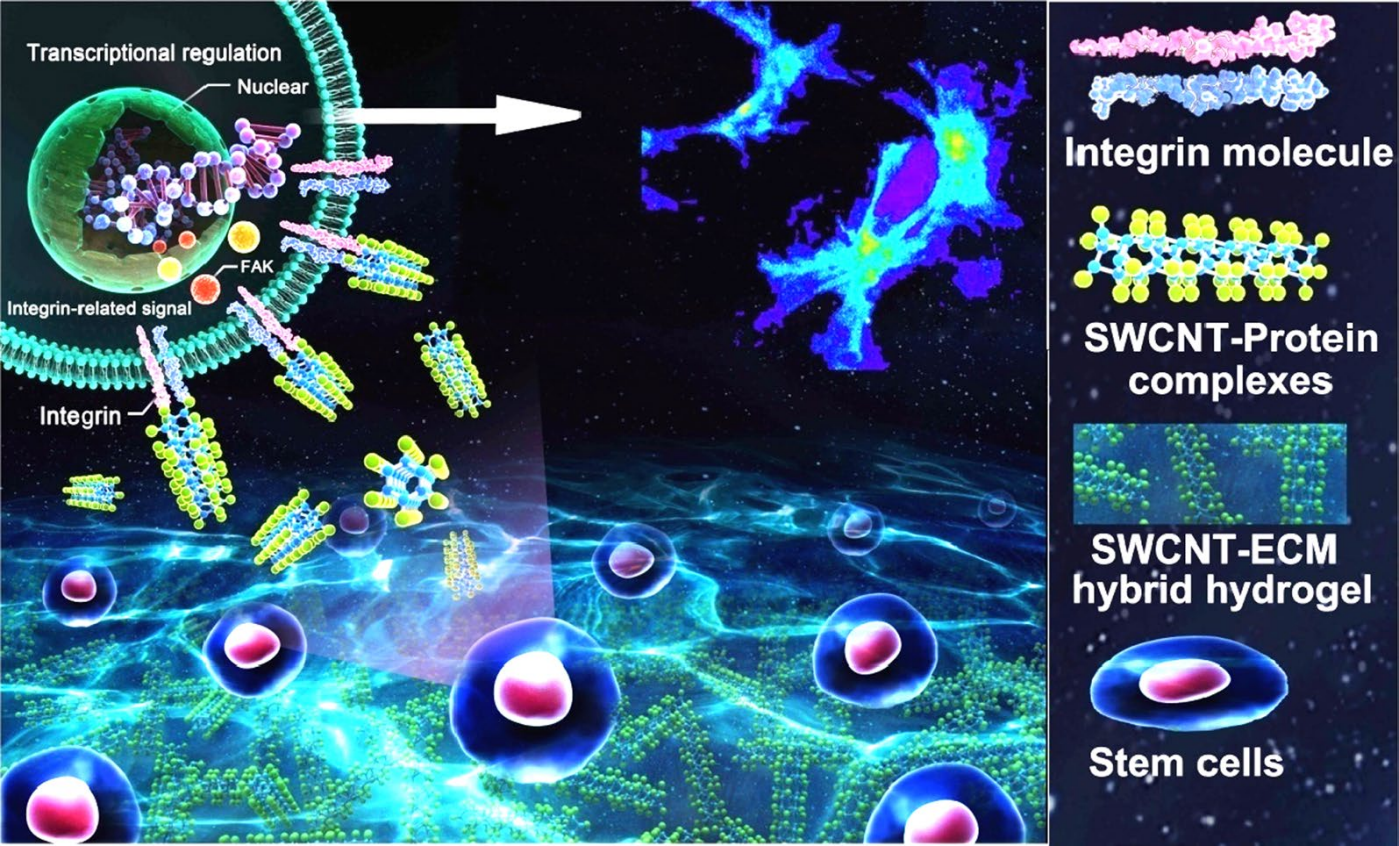
Regulation of SWCNT-ECM hybrid hydrogels on seeding cell fate and the underlying mechanism

Herein, a hybrid hydrogel (HH) was developed by incorporating single-wall carbon nanotubes (SWCNTs) into heart-derived ECM. Through interaction with ECM proteins, insoluble SWCNTs were well dispersed, resulting in functional niches in HH which could act like biophysical cues to activate the integrin-related pathways (Scheme 1). The efficacies of the HH in facilitating cardiac-lineage development of seeding cells in vitro and in vivo were systematically evaluated.

## Materials and methods

### Preparation and characterization of SWCNT-ECM hybrid hydrogels

Decellularized heart ECM was prepared with the same procedures as our previous report [26]. SWCNT-ECM hybrid hydrogels (HH) were prepared by adding SWCNTs (diameter 0.8–1.2 nm; length 100–1000 nm, US nanomaterials research.) at different final concentration. The SWCNT-ECM solution was well mixed by stirring. For gelation, pH values of solution were adjusted to 7.4 with sodium hydroxide (NaOH).

The detailed protocols for heart ECM preparation and HH characterization were available in supporting information.

### Cell culture and in vitro evaluation

Cardiomyocytes were isolated from neonatal rats and cultured with DMEM supplemented with 10 % FBS (fetal bovine serum, Gibco). HHs with different SWCNT concentration (0, 0.5, 1 and 2 mg/mL) were evaluated for supporting the adhesion and survival of cardiomyocytes. The detailed procedures were described in supporting information.

Brown adipose tissues were obtained from scapula of young SD rats (Male, 80–100 gram). Brown adipose-derived stroma cells (BADSCs) were isolated with the same procedures as previously described [27]. The influence of HH on the adhesion and survival of BADSCs were evaluated with the similar methods as described above for cardiomyocytes. Adhered cells per field were counted under phase contrast microscope. The cell spreading area and protuberance were analyzed using Image-Pro Plus software. Cell viability was evaluated by Live/Dead staining (L34951, Thermo Fisher Scientific, USA) and Alamar Blue assay respectively according to manufacturers’ instruction. Cell proliferation was assessed by Brdu staining. Briefly, Brdu was added into cultured cells according to the kit instruction (abcam) and incubated for 1 h. Cells were fixed with 4% paraformaldehyde. Fixed cells were treated with 0.3% Triton for 30 min and then, 2 M HCl was used to treat cells for 15 min followed by boric acid treatment for 15 min. After washing three times with PBS, 10% horse serum was added and incubated for 1 h. Anti-BrdU antibody was added according to the manufacturer’s guidance and incubated overnight at 4℃. The corresponding secondary antibodies were added and incubated for 2 h at room temperature. The nuclei were stained by DAPI.

### Real time PCR and Western Blotting

For real-time PCR detection, cells were collected (n = 3 per group) and lysed with Trizol (Invitrogen). Total RNA was extracted isopropanol precipitation method according to the standard procedure. cDNA was synthesized by reverse transcription using a commercial kit (TianGen) according to manufacturer’s instruction. Quantitative real-time PCR was then performed using SYBER Green Master Mix (Takara, Dalian, China). The gene-specific primers were available in supporting information. Gene expression was quantified with the 2^−ΔΔCt^ method.

For western blotting analysis, 3 independent samples (n = 3 per group) which were prepared under the same conditions were collected and mixed for the next assay. Cells were lysed using Laemmli Sample Buffer (Bio-Rad) for protein extraction. The protein was quantified with BCA protein assay kit (Pierce, Thermo Scientific, USA). Protein electrophoresis was performed with 10% SDS-containing polyacrylamide (SDS-PAGE) gel. The details for western blotting were available in supporting information.

### Calcium transient measurements

Intracellular calcium ion current was detected using Fluo-4 AM kit (Invitrogen) according to the manufacturer’s instruction and the previous report [26]. Briefly, cultured cells were washed two times with Tyrode’s solution. Then 10 mM fluo-4 AM working solution was added and incubated with cells for 30 min at 37 ℃. Fluo-4 AM was removed and cells were washed with Tyrode’s solution for 3 times. A confocal laser scanning microscope (Nikon Eclipse Ti-E) was employed to catch the fluorescent signals at the exciting light of 488 nm and detecting light of 505 nm. Data was analyzed with Volocity software (Nikon).

### Preparation of 2D and 3D cardiac tissues

To prepare 2D cell sheet, 1 cm × 1 cm sized glass slides were used and coated with HH. Briefly, glass slides were firstly treated with concentrated sulfuric acid followed by extensive washing with sterile water. Then, the slides were immersed in ethanol for 1–2 h. After air dry naturally, 150 µL HH solution was added onto slides uniformly. The slides were dried at 37 ℃ and a thin layer of HH substrate would be formed on the slide. Before cell seeding, the slides were placed into 24 well plate, 5 × 10^4^ cells in 0.5mL DMED/10% FBS medium were seeded onto one slide and cultured at 37 ℃, 5% CO_2_ incubator.

To construct 3D cardiac tissue, the previously reported method was referenced [4]. Briefly, 2% agarose solution was added into 24 or 48 well plate (300 µL/well for 48 well plate and 500 µL/well for 24 well plate). After solidification, capillary glass tubes of suitable length were inserted into agarose gel for fixing cardiac tissues during culture. HH solution was mixed with 2 × α-MEM at the ratio of 1:1. Then, BADSCs were suspended in HH solution at the concentration of 2.5 × 10^6^/mL and pH was regulated to about 7.4. The cell suspension was poured into 24 (200 µL/well) or 48-well (100 µL/well) plates. The plate was placed into 37 ℃, 5 % CO_2_ environment for gelation. α-MEM supplemented with 10% FBS was used for cultivation.

### Animal experiment

All animal experiments were conducted according to the Guide for the Care and Use of Laboratory Animals and approved by the Institutional Animal Care and Use Committee (IACUC) of Beijing institute of basic medical sciences (Beijing, China).

Adult male Sprague Dawley (SD) rats (200 g ± 10 g) were used. Myocardial infarction was prepared by the ligation of the left coronary artery as previously reported [26, 28]. Then, animals were randomly divided into four groups: (1) control group: intramyocardial injection of 100µL PBS; (2) HH-treated group: intramyocardial injection of 100 µL HH solution; (3) BADSC-treated group: intramyocardial injection of 5 × 10^6^ BADSC in 100 µL PBS; (4) HH + BADSC-treated group: intramyocardial injection of 5 × 10^6^ BADSC in 100 µL HH solution. Cell injection was performed with the same protocol as previously reported [26]. For in vivo tracking, cells were labeled with DiI dyes before transplantation according to manufacturer’s instructuion (Invitrogen).

Multimodality of methods were employed for evaluating therapeutic outcome, including PET/CT imaging, echocardiogram, histology and immunohistochemistry. Details of each method were available in additional file.

### Statistical analysis

All data were expressed as mean ± SD. GraphPad Prism 7 software was employed for mapping and statistical analysis. *Student’s t*-test was used for data comparison between two groups, while one-way ANOVA with Tukey’s post-hoc test was used for data comparison of more than two groups. *p* < 0.05 was considered as statistically significant.

## Results and discussion

### Preparation and characterization of SWCNT-ECM hybrid hydrogels

**Fig. 1:**
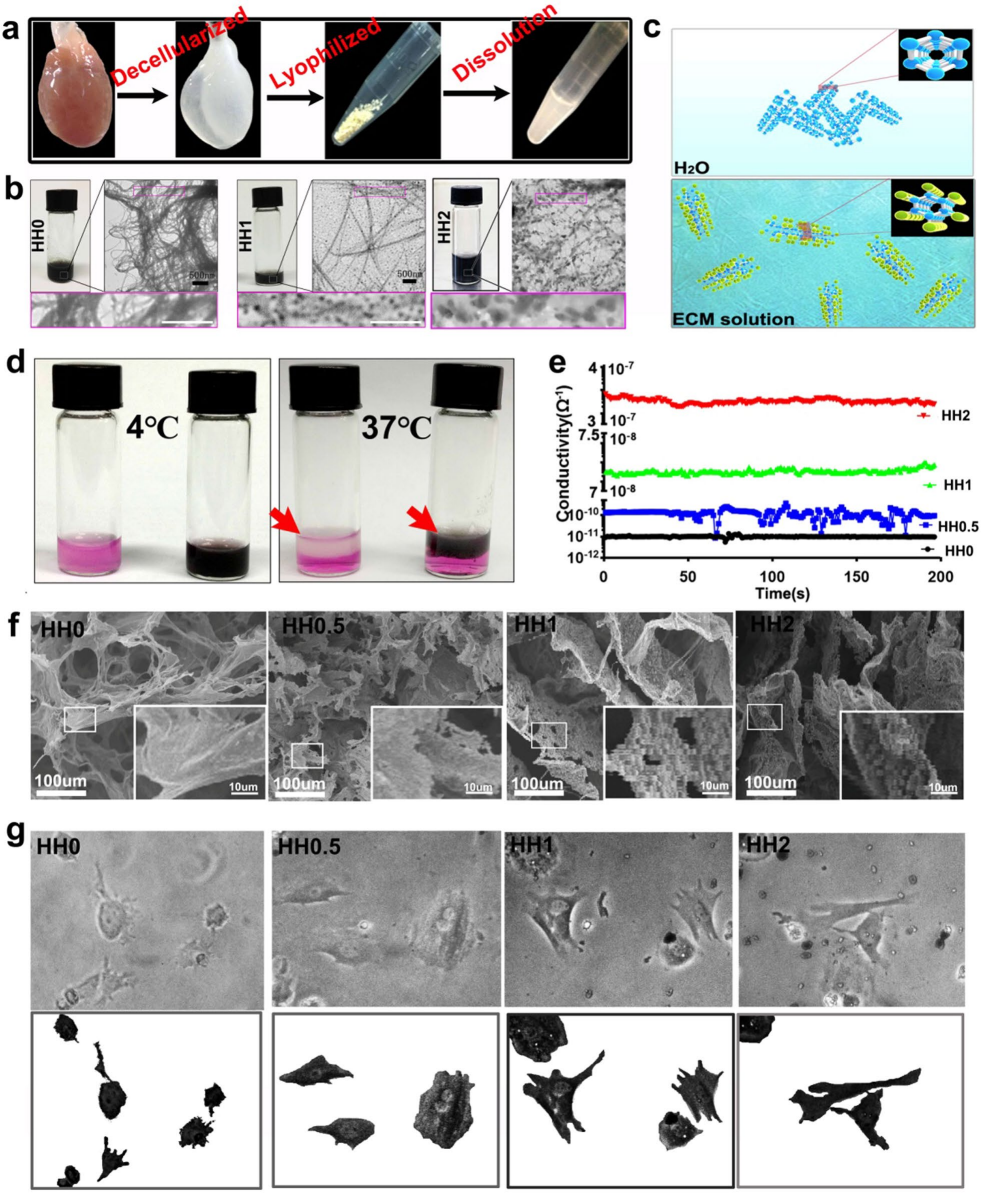
Preparation and characterization of HHs. **a **The procedure of preparing decellularized heart ECM; **b** TEM observation of SWCNT and HH solution; **c** the proposed scheme of SWCNT dispersed in HH solution through interacting with ECM; **d** gelation of ECM hydrogel and HH at 37 ℃; **e** the electrical conductivities of HHs containing different concentration of SWCNTs; **f** SEM observing the surface morphology of HH; **g** adhesion and extension of cardiomyocytes on HH-coated substrate

With the established method [29], decellularized ECM was prepared using adult rat hearts. Then, SWCNT-ECM HHs were prepared by incorporating SWCNT of different doses into ECM hydrogels (HH0: pure ECM hydrogel; HH0.5: HH containing 0.5 mg/mL SWCNT; HH1: HH containing 1 mg/mL SWCNT; HH2: HH containing 2 mg/mL SWCNT). The preparation procedure was demonstrated in Fig. 1a. Previously, both SWCNT and multi-walled carbon nanotube (MWCNT) were investigated for biomaterial modification. However, due to the different structures, there are distinct properties between them. The study selected SWCNT instead of MWCNT mainly considering the following factors: (1) Size. SWCNTs are made up of a single graphene sheet rolled-up, whose diameters usually range from 0.4 to 2 nm; while MWCNTs are made up of several concentric graphene cylinders, whose diameters could range from 1.4 to 100 nm [30, 31]. Apparently, the diameter distribution of SWCNTs is much narrower and modification with SWCNTs may produce more homogenous niches in composite materials. In addition, due to the unidimensional structure, SWCNTs have high surface area which is beneficial for interacting with ECM proteins [32]; (2) Electrical property. SWCNTs have better electron-transfer property that very low content is effective to enhance the conductivity of composite materials [33–36]. The conductivity was especially important for those scaffolds used in cardiac or neural tissues due to their electrical activities. In previous reports, SWCNTs were frequently selected to modify scaffolds for cardiac or neural application [31, 37–41]; and (3) Mechanical property. SWCNTs are of higher tensile strength and elastic modulus, more suitable for soft tissue scaffolds; while MWCNTs have higher rigidity, more suitable for stiff tissue scaffolds, such as bone [42, 43]. In fact, MWCNTs were often incorporated into composite scaffolds to promote osteogenesis [44–46]. Collectively, SWCNTs were selected in the study.

It was known that SWCNT was insoluble and usually aggregated in water (Fig. 1b). Surprisingly, when SWCNTs were mixed with ECM solution, they could be well dispersed without aggregates. We supposed that it may be due to the interaction between SWCNTs and ECM proteins. Lots of proteins may be adsorbed on SWCNTs due to hydrophobic interaction, covering the hydrophobic surface region on them and thus promoting their dispersion in water, as illustrated in Fig. 1c.

It was reported that ECM hydrogel was thermo sensitive and would gelatinize under 37 ℃, neutral pH conditions [29]. In the study, the concentration of ECM solution for gelation was optimized as 6 mg/mL (Additional file 1: Figure S1). By comparison, it was found that the rheological and gelation properties were similar before and after SWCNT incorporation (Fig. 1d). Then, the mechanical properties of various HHs were evaluated. Compared with ECM hydrogel, the compressive modulus of hydrogels was significantly enhanced after SWCNT incorporation, indicating the strength and bearing capacity of hybrid hydrogels was improved (Additional file 1: Figure S2). As is known, heart is a pulsating organ which requires a good bearing capacity for the ECM [47, 48]. Therefore, we supposed that such mechanical improvement in HH by SWCNT was beneficial for application in myocardium. Another physiochemical property, the conductivity of hydrogel, was also significantly enhanced by SWCNT in a dose-dependent manner (Fig. 1e). The electrical activity was a key characteristic of myocardium, which was indispensable for cell-cell communication and synchronous beating formation [49]. Due to the improved conductivity, it could be supposed that the hybrid hydrogels may possess a better performance in regulating cardiac seeding cells. Next, SEM observation demonstrated the microroughness of the scaffolds was significantly enhanced after SWCNT incorporation (Fig. 1f).

### The influence of HH on adhesion and development of primary cardiac cells

Previously, carbon nanotubes have been employed to improve the properties of bio-scaffolds. It was revealed that cell attachment and spreading on carbon nanotubes would be facilitated by integrin binding [50, 51]. The mechanism may be related to the improved surface topography of scaffolds, such as surface microroughness [52]. The increased microroughness of scaffolds would promote the extension of cell antennae, the expression of integrin (especially integrinβ1) and thus enhance cell adhesion [52–54]. On the basis, primary cardiac cells were used for evaluation. HHs containing different dose of SWCNTs were used to coat culture plates. It was demonstrated that both adhered cell number and cell extension areas were increased in HH-coated plates compared with ECM hydrogel-coated ones (HH0, Fig. 1g). The quantitative analysis showed that it was optimal for cell adhesion and extension when the concentration of SWCNT was 1 mg/mL (HH1). Further increase of SWCNT concentration led to some decrease of cell adhesion and extension (Additional file 1: Figure S3). This may be due to the cytotoxicity of high dose of SWCNTs [55]. During the cultivation, the least cell death rate was also observed in HH1-coated plates (Additional file 1: Figure S4).

**Fig. 2:**
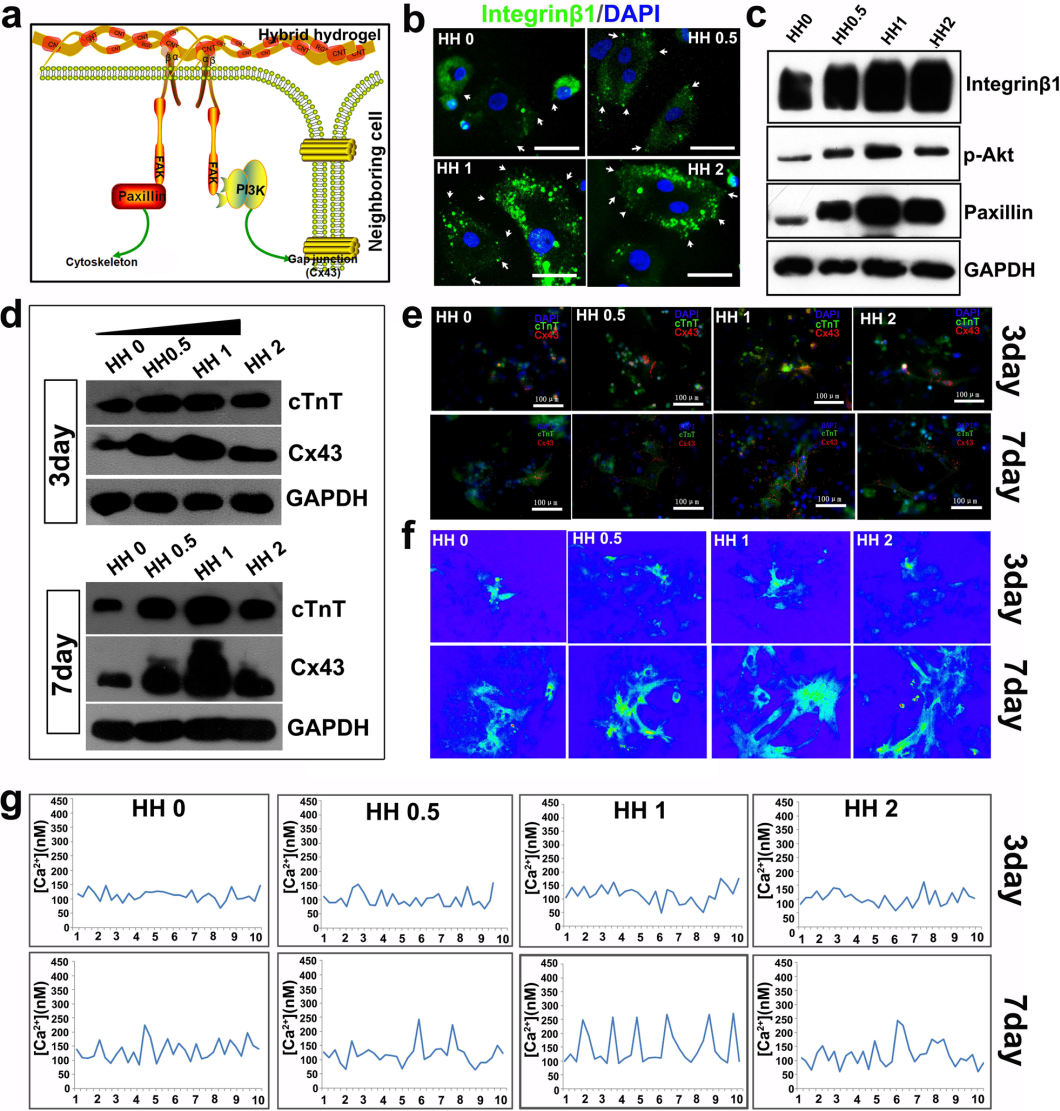
Effects of HH on cardiomyocytes and the underlying mechanism. **a** The proposed mechanism of HH regulating cardiomyocytes through integrin β1-related pathway; **b** immunostaining of cardiomyocytes growing on HH using anti-integrin β1 antibody; **c** detection of signal molecules downstream integrin β1 with western blotting; **d** western blotting of cardiac markers in cells growing on HH; **f** imaging of calcium transient using Fluo-4 AM; **g** Intracellular calcium ion current of cardiomyocytes growing on HH

As integrin family proteins play important roles in cell adhesion [54, 56, 57]. We supposed that integrin and the downstream pathways may be activated in HH (Fig. 2a). 6 h after adhesion on HH-coated plate, the upregulation of integrinβ1 in cardiac cells was verified by immunostaining (Fig. 2b**).** Then, western blotting provided additional evidence for the activation of integrin-related pathways (Fig. 2c). It was also known that integrin-related pathways played important roles in regulating cell fate and establishment of cell-cell communication, which were essential for the development of functional cardiac tissues (Fig. 2a) [58, 59]. cTnT (cardiac marker) and connexin43 (gap junction marker, CX43) were detected to evaluate the structural development of cardiac cells. As shown in Fig. 2d, western blotting showed that cTnT at day 7 and CX43 at both day3 and day7 were upregulated in cells growing on HH-coated plates, especially in HH1-coated ones. The cells were further co-stained by anti-cTnT and CX43 antibodies to determine their co-localization (Fig. 2e). It could be seen that there were apparently more co-stained areas in cells growing on HH-coated plates, indicating that HH promoted the maturation and gap junction formation of cardiac cells. Calcium imaging was then performed to evaluate the functional development of cardiac cells. As shown in Fig. 2f and g, compared with ECM hydrogel (HH0), HH significantly promoted the development of rhythmic calcium activities in cardiac cells, especially HH1. Calcium activity played important roles in the cell-cell excitation transduction of mature cardiomyocytes. Thus, the results indicated that the HH promoted the functional mature of these cells. The underlying mechanism may be complicated. One of the potential mechanisms should be related to the activation of signal pathways promoting cell development by SWCNTs. Previously, it has been reported that SWCNTs could activate several pathways influencing the cell fates, such as ERK and RhoA pathways [60, 61]. Another potential mechanism may be related to the improved ECM property by SWCNTs. Conductivity is such a key property. Several studies have confirmed that conducting scaffolds could promote neural or cardiac development of seeding cells through facilitating cell–cell communication [62–65]. Therefore, in the study, the improved conductivity of HH due to the incorporation of SWCNTs should be beneficial for cardiac cell development. Of course, other unrevealed mechanisms could also exist, which deserved in-depth investigation in the future.

### The influence of HH on brown adipose-derived stem cells

**Fig. 3:**
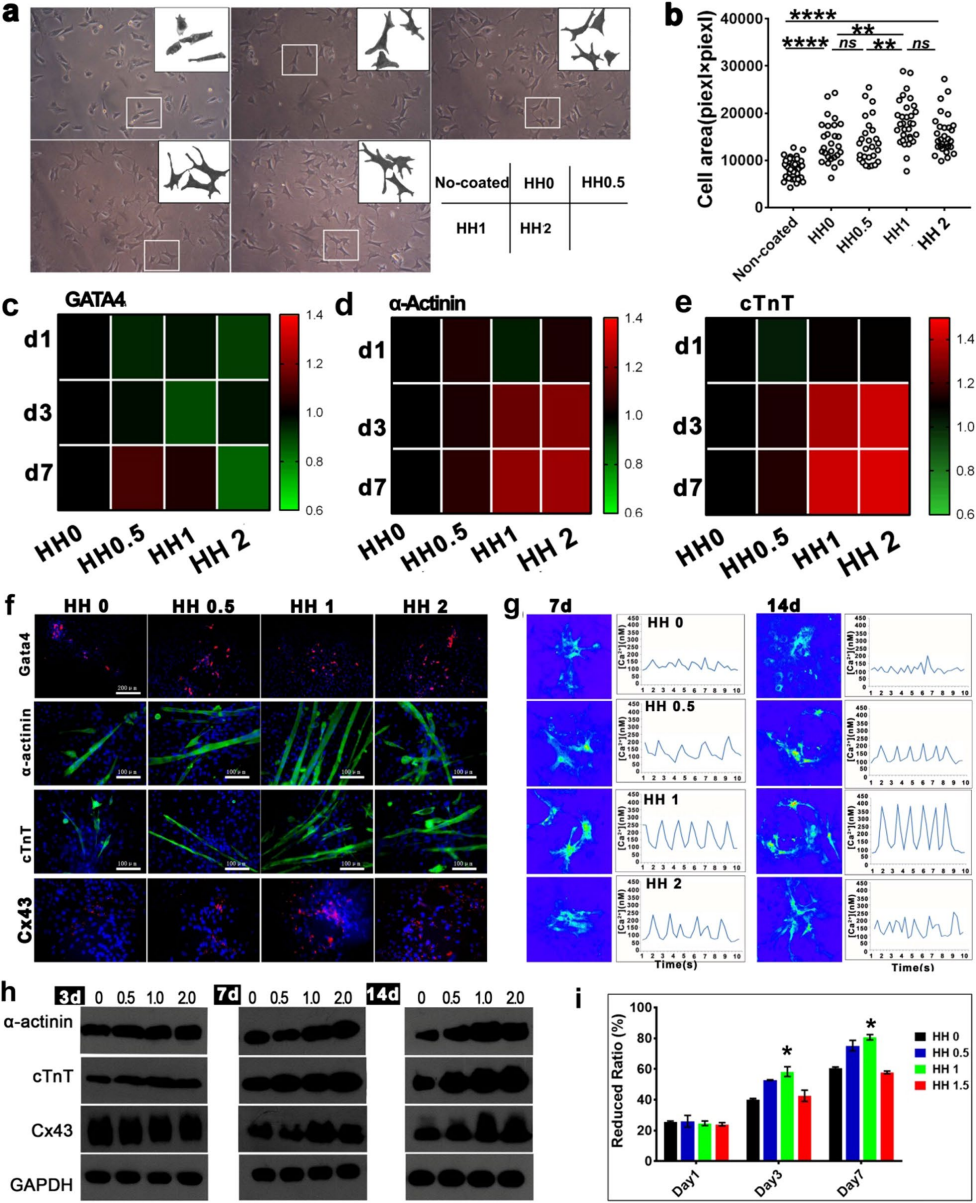
The adhesion, survival and differentiation of BADSCs on HHs. **a** The morphology of BADSCs on HHs; b, the spreading area of BADSCs on HHs; **c**–**e**, thermography of cardiac genes in cells growing on HH-coated substrate; **f** cardiac differentiation of BADSCs on HH-coated substrate; **g** Intracellular calcium ion current of BADSC-derived cardiomyocytes growing on HH-coated substrate; **h** western blotting detecting the expression of cardiac markers in BADSCs growing on HH-coated substrate; **i** the prolifieration of BADSCs on HH-coated substrate by Alamar Blue Assays.(*p < 0.05; **p < 0.01; ***p < 0.001)

Stem cells were great seeding cells in cardiac tissue engineering and regeneration. Among various stem cells, mesenchymal stem cells (MSC) were the mostly investigated ones due to their low immunogenicity [66]. At present, adipose tissue was found as the most abundant resource of MSC and adipose-derived MSCs were considered clinically promising in future [67]. However, the MSC was of low cardiomyogenic potential. In recent years, brown adipose-derived stem cells (BADSC) were found with high cardiomyogenic potential [27, 68]. Importantly, BADSCs could spontaneously differentiate into cardiomyocyte-like cells with rhythmic beating, which has been observed by independent groups [27, 68, 69]. It was considered that BADSCs may be better candidate cells for myocardial repair than MSC. Therefore, BADSCs were employed in the study for further evaluating HHs. BADSCs were isolated and characterized as Additional file 1: Figure S5. Like that for cardiac cells, HH significantly promoted BADSC adhesion and extension compared with pure ECM. The optimal HH was HH1 (Fig. 3a and b, Additional file 1: Figure S6a). Meanwhile, the tentacle outgrowth from cells was significantly more on HH-coated plates (Additional file 1: Figure S6b). No difference of cell viabilities was observed at day1 among different groups, but significant higher viability was maintained in HH1 treated group (Additional file 1: Figure S7–S9) at day 3. The result indicated a good supporting of the HH (especially HH1) for BADSCs.

Cardiac markers, including early stage marker gata4 and mid-late stage markers cTnT and α-actinin, were detected at gene and protein levels to determine the effects of HH on BADSC differentiation. RT-PCR showed that Gata4 expression was similar among cells growing on different hydrogels, while cTnT and α-actinin were significantly upregulated by HHs. The optimal effect was achieved in HH1 and HH2 (Fig. 3c–e and Additional file 1: Figure S10). The results indicated that the HHs mainly promoted the mid-later stage differentiation of BADSCs to cardiomyocytes. The expression of Gata4, α-actinin and cTnT from immunostaining was consistent with those from RT-PCR. No significant difference of early-stage marker Gata4 was observed among different groups, while significantly more α-actinin and cTnT cells were detected in those growing on HHs (Fig. 3f). The gap junction protein CX43 in differentiated BADSCs was also upregulated by HHs, indicating HHs promoted the formation of structural connection between stem cell-derived cardiomyocytes (Fig. 3f). This would be beneficial for the functional development and synchronous beating formation of BADSC-derived cardiomyocytes. On the basis, calcium transients were imaged and demonstrated that HHs significantly promoted the rhythmic calcium transients in BADSC-derived cardiomyocytes (Fig. 3g). The effects were also correlated to the concentration of SWCNT, and the optimal effect was observed in HH1. The attenuated effects of higher-concentration SWCNT (HH2) may be due to the cytotoxicity as mentioned above. Then, western blotting was performed to determine cardiac markers at protein level, which provided consistent evidence (Fig. 3 h). Brdu labeling demonstrated a significant promotion effects of HH on BADSC proliferation (Additional file 1: Figure S11 and Fig. 3i), and the effects were correlated with SWCNT concentration. The optimal concentration was also 1 mg/mL.

### **Construction of 2D and 3D cardiac tissues with rhythmic beating using HH and adult stem cells**

Engineered cardiac tissues were considered as potential substitutes for heart transplantation [70, 71]. In the past years, independent groups have explored the construction of engineered cardiac tissues, including cardiac ring, cardiac patch and cardiac microsphere [72–74]. However, all of these cardiac tissues with rhythmic beating were constructed using primary cardiomyocytes or ESC/iPSC-derived cardiomyocytes. Cardiac sheets constructed using adult stem cells were previously investigated too by independent groups, and they were applied *in vivo* for myocardial repair [75–77]. However, to date, it has not been reported to construct an cardiac tissue with the rhythmic beating using adult stem cells. Based on the potent regulation of HH1 on the cardiac differentiation and maturation of BADSCs, we explored the construction of a functional myocardium with adult BADSCs as seeding cells and HH1 as scaffolds. 2D myocardial sheet and 3D myocardial tissue were constructed respectively. 2D myocardial sheet was constructed by seeding a monolayer BADSCs onto HH1 membrane (Additional file 1: Figure S12a). After 4 days’ culture, visible beating to the naked eyes was present. With time, the beating was more stable and powerful. After 8 days’ culture, 70% (7/10) cell sheets were observed with rhythmic beating (Additional file 1: Figure S12b, Additional file 2: Move S1). However, no visible beating was found in control sheets which were constructed using BADSCs and pure ECM. 3D myocardial tissues, constructed with the previous reported method (Additional file 1: Figure S12c) [28], were also observed with rhythmic beating after 6 days’ culture (6/10), which was more powerful than 2D cardiac sheet (Additional file 1: Figure S12d, Additional file 3: Move S2). No visible beating was found in control tissues (constructed using BADSCs and pure ECM hydrogel), though local beating of tissues could be observed under microscopy (Additional file 4: Move S3). Histological examination showed more aligned distribution of BADSCs in beating 3D tissues compared with control tissues (Additional file 1: Figure S13), which may explain the formation of synchronous beating. The successful construction of rhythmic beating cardiac tissues suggested that the HH could provide a more potent niche than pure ECM to promote the structural and functional development of seeding cells towards cardiac tissues.

### The influence of HH on BADSC engraftment in infarct myocardium

**Fig. 4:**
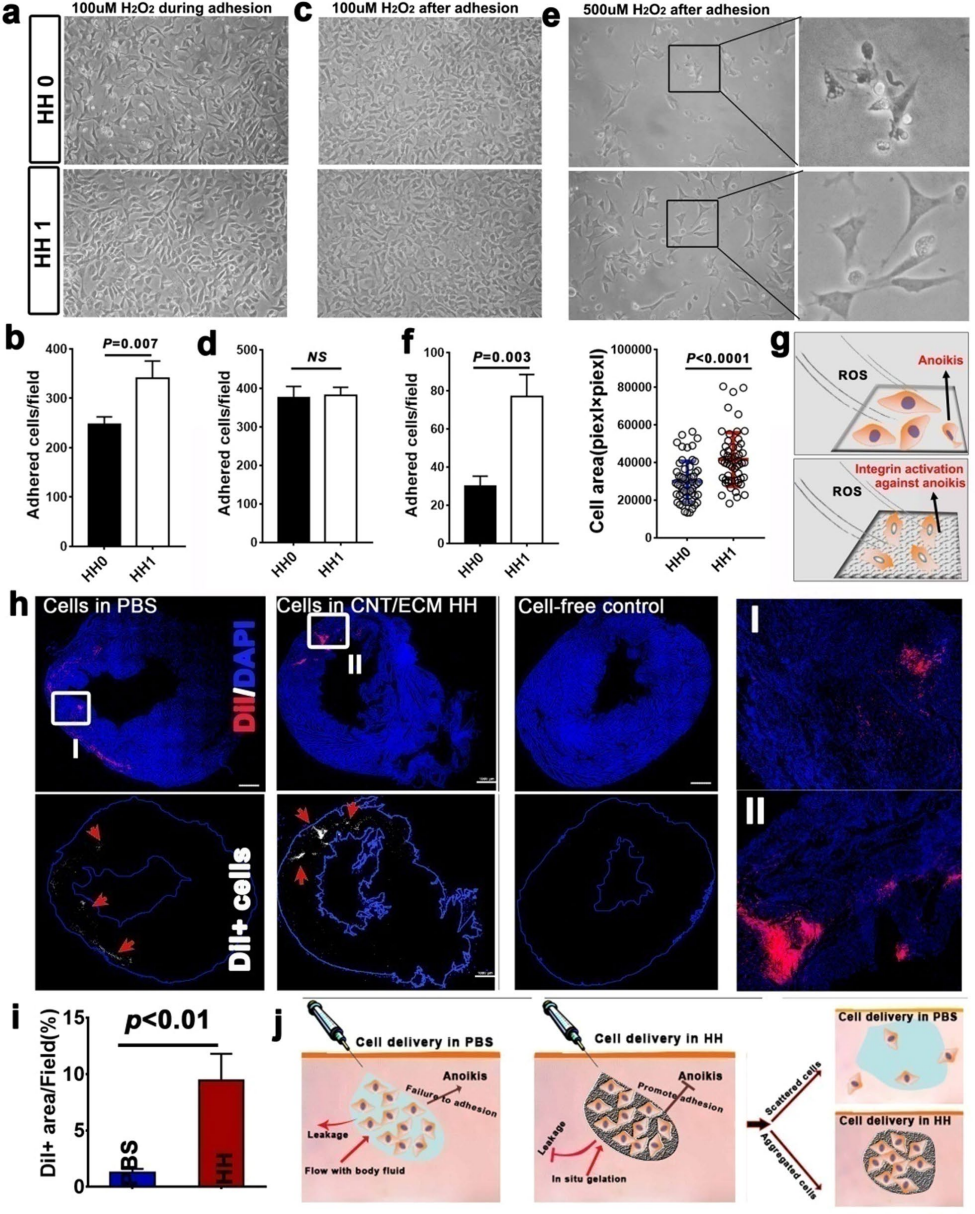
HH protects BADSCs against anoikis in vitro and in vivo***.*** **a** HH promoted the adhesion of BADSCs in vitro against ROS (H_2_O_2_ was used to provide ROS); **b**, **c** HH protected BADSCs in vitro against ROS after adhesion; **d** schematic illustrating the protection of HH on BADSCs against ROS; **e** quantitative analysis of adhered cells on different substrate under treatment of 100 µM H_2_O_2_ for 6 h. H_2_O_2_ was added immediately after cell seeding; **f **quantitative analysis of adhered cells on different substrate under treatment of 100 µM H_2_O_2_ for 6 h. H_2_O_2_ was added after cell adhesion (12 h after cell seeding); **g** analysis of cell adhesion and extension under treatment of 500 µM H_2_O_2_ for 6 h. H_2_O_2_ was added after cell adhesion (12 h after cell seeding); **h** In situ retention of BADSCs after intramyocardial injection in PBS and in HH; h, schematic illustrating the mechanism of cell retention in situ after delivery in HH; **i** Quantitative analysis of cell retention in recipient

Myocardial ischemia was accompanied with large generation of ROS [78], which may lead to the anoikis (caused by the failure of adhesion to surrounding matrix) of transplanted cells. Thus, the efficacy of HH1 protecting BADSC under ROS against anoikis was evaluated in vitro during and after cell adhesion. When 100 µM H_2_O_2_ was added immediately after cell seeding, significantly more BADSC adhered on HH1-coated matrix than that on HH0-coated one after 6 h (Fig. 4a and b). However, no significant difference of that was observed between HH1 and HH0 if H_2_O_2_ treatment was performed after cell adhesion (Fig. 4c and d). The dose of H_2_O_2_ was then increased to 500 µM. It was observed that lots of BADSCs shrank and fell off HH0-coated plate after 12 h, which, however, was significantly attenuated in cells growing on HH1 (Fig. 4e). In addition, both the number of adhered cells and their spreading area were significantly decreased for cells growing on HH0-coated plates compared with those growing HH1-coated ones (Fig. 4f**)**. On the basis, a scheme was proposed for HH1 promoting BADSC adhesion under ROS condition (Fig. 4g**)**. To evaluate the in vivo efficacy of HH facilitating myocardial repair, HH1 was employed as an injectable scaffold for intramyocardial delivery of BADSCs. 1 week after myocardial delivery, the engraftment of BADSCs (DiI labeling) in recipient myocardium was evaluated. Significant more DiI-positive cells could be seen from hearts receiving HH1 plus BADSCs injection than those receiving BADSCs alone injection (BADSCs in PBS, Fig. 4h and i). We supposed that enhanced cell retention due to gelation, and promoted cell adhesion should be the main reasons for improved engraftment of BADSCs by HH (Fig. 4j).

### Structural and functional outcomes after intramyocardial delivery of BADSCs in HH

**Fig. 5:**
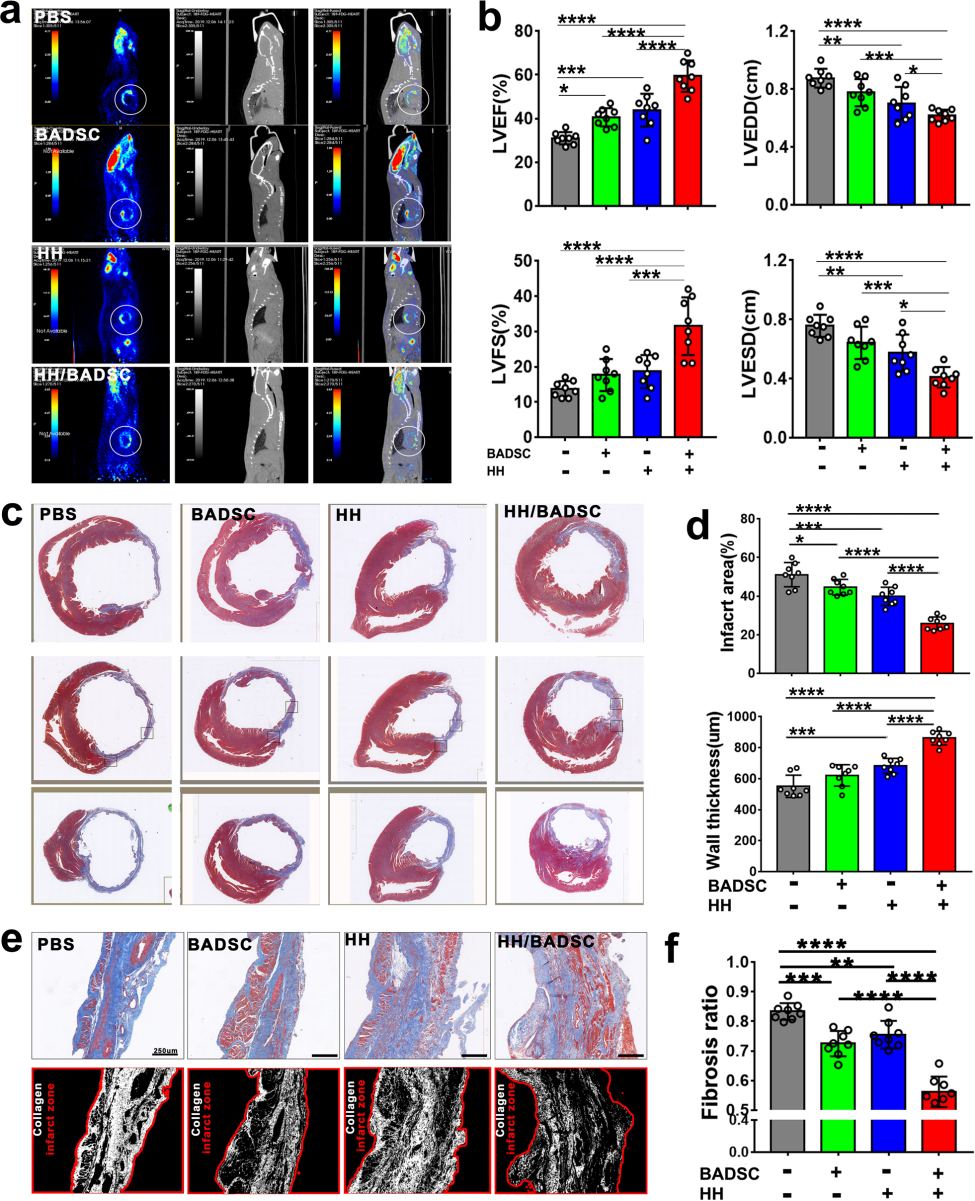
Structural and functional analysis of ischemic hearts. **a** PET/CT imaging detecting the infarct size of ischemic hearts in living animals; **b** echocardiogram detecting the heart function. The functional parameters, LVEF, LVFS, LVEDD and LVESD were acquired; **c** Masson trichrome staining demonstrated the morphology and structure of infarct hearts; **d** Comparison of infarct size and left-ventricle wall thickness of ischemic hearts receiving different treatment; **e** the fibrosis of infarct area was visualized after Masson trichrome staining and recognized using Image Pro Plus software; **f** myocardial fibrosis was statistically analyzed among different groups

The structure and function of ischemic hearts were evaluated by non-invasive PET/CT imaging and ultrasoundcardiogram respectively. Infarct area with low myocardial viability could be apparently seen in hearts from PET/CT imaging (Fig. 5a). Compared with PBS control, HH or BADSCs treatment significantly improved myocardial viability that smaller infarct area was observed, while the greatest improvement in myocardial viability was detected in rats treated by HH plus BADSCs. The functional evaluation by ultrasoundcardiogram achieved consistent results that the functional parameters, including LVEF, LVFS, LVEDD and LVESD, were significantly improved by HH and BADSCs, HH plus BADSCs resulted in the greatest improvement (Fig. 5b). After sacrifice, the hearts were explanted and infarct size was determined on Masson trichrome stained sections. It was shown that the infarct size was significantly decreased in HH and BADSCs-treated animals, while left ventricle wall thickness was significantly increased compared with PBS-treated control animals (Fig. 5c and d). The best improvement was also seen in animals treated by HH + BADSCs. Histological analysis was further performed to determine the fibrosis in infarct myocardium among different groups. The fibrotic ratios in infarct zone were significantly decreased in HH and BADSCs treated groups compared with PBS-treated one, and combination of HH with BADSCs resulted in further improvement of myocardial fibrosis (Fig. 5e and f). The residual of SWCNT in host myocardium was determined too on heart sections and none was observed (Fig. 5c), indicating SWCNT may have been completely degraded. Previously, the biodegradability of carbon-nanotube has been reported by different studies. In vivo, NADPH oxidase was found to play important role in SWCNT degradation. Macrophages were the key cells during NADPH oxidase-dependent SWCNT degradation [79]. It was revealed that SWCNT would be degraded by approximately 25–30% within first 4 days after macrophage uptake [80]. The safety of SWCNT was also reported previously. Two factors would attenuate the toxicity of SWCNT: protein binding and bio-degradation [80, 81]. In the study, SWCNT was bound with abundant ECM proteins in HH. Meanwhile, the ischemic myocardium may provide a beneficial environment for SWCNT degradation, as lots of activated macrophage existed [82]. The data suggested that the in vivo application of SWCNT in the study should be safe.

Then, the vascularization was determined on anti-vWF-immunostained sections, which achieved consistent results that the greatest revascularization was observed in hearts receiving HH + BADSC treatment (Additional file 1: Figure S14).

### **The influence of HH on cardiac differentiation of BADSC*in vivo***

To evaluate the in vivo development of transplanted BADSCs, heart sections were immune-stained with cardiac markers cTnT and α-actinin. According to the expression of cardiac markers as well as their morphologies, engrafted BADSCs (DiI+) were divided into three types: (1) undifferentiated cells (UD); (2) cardiac differentiation but unmatured cells (UM); (3) cardiac differentiation and relative matured cells (RM).

**Fig. 6:**
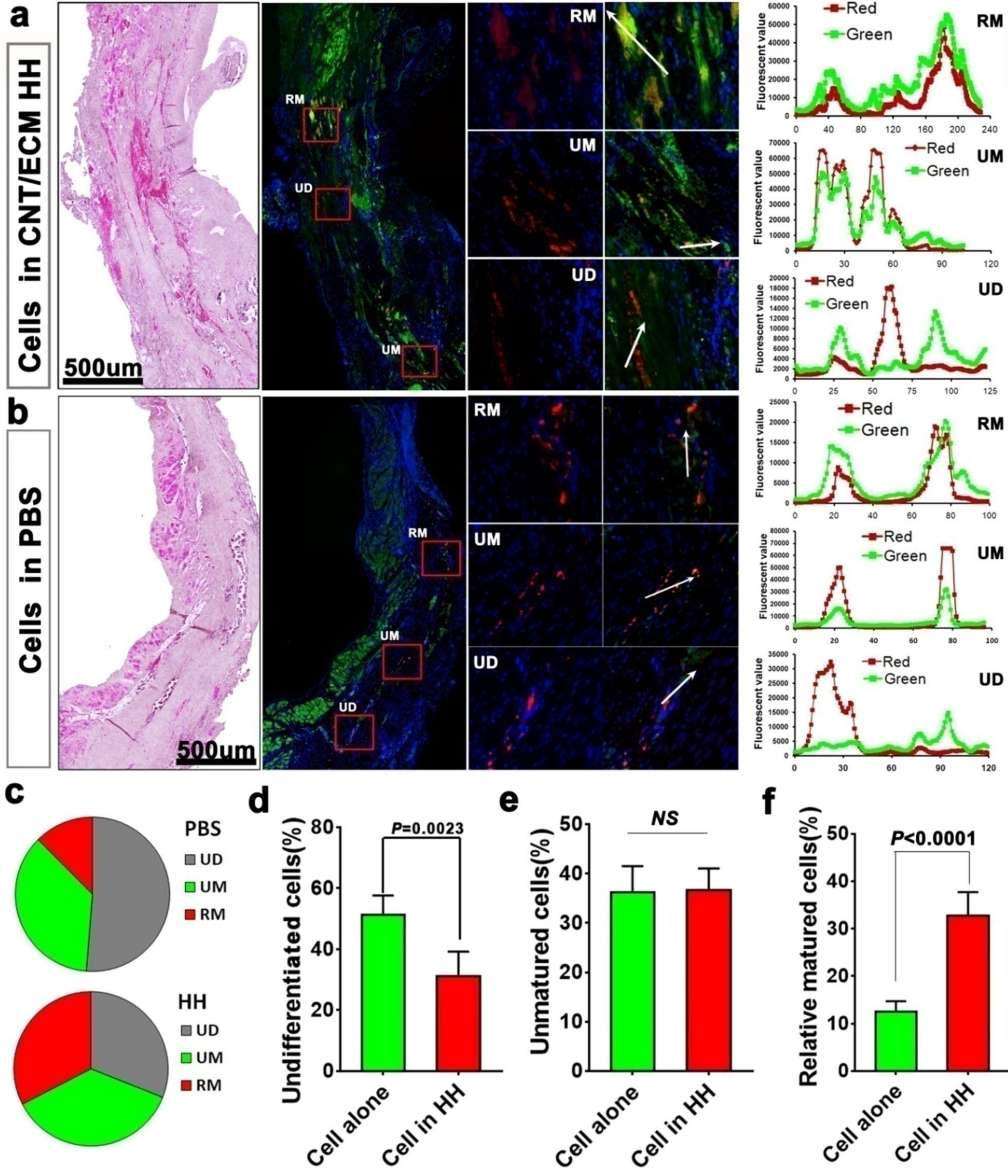
In situ differentiation of BADSCs after intramyocardial delivery in HH. **a** Engraftment and cardiomyogenic differentiation of BADSCs in recipient myocardium after delivery in HH; **b** engraftment and cardiomyogenic differentiation of BADSCs in recipient myocardium after delivery in PBS; **c** the ratio of engrafted BADSCs at different stages of differentiation; **d**–**f** quantitative analysis of engrafted BADSCs at different stages of differentiation. UD indicates undifferentiated; UM indicates unmatured; RM indicates relatively mature

As shown in Fig. 6a and Additional file 1: Figure S15, co-localization of DiI and cardiac markers were detected both in BADSC alone and HH + BADSC groups. Quantitative analysis showed that undifferentiated cells were significantly less in HH + BADSC group than that in BADSC alone group (31.2 ± 8% VS 51.3 ± 6.4%, *P* < 0.01; Fig. 6c and d). In other word, more cells differentiated into cardiac lineage in HH + BADSC group. However, among the differentiated cells, no significantly difference of UM cells was detected between BADSC alone and HH + BADSC groups **(**36.6 ± 4.4% VS 36.6 ± 5.3%; Fig. 6e**)**, while significantly higher ratio of RM cells was detected in HH + BADSC group **(**32.8 ± 5% VS 12.5 ± 2.3%, *P* < 0.001; Fig. 6f**)**, indicating that HH mainly promoted the mid-late stage differentiation of BADSC towards cardiac lineage, which was consistent with the in vitro results. These data provided in vivo evidence that a niche was formed in SWCNT-ECM HH which potently facilitated tissue-specific development of seeding cells toward cardiac lineage. Furthermore, it could be concluded that more regenerated cardiomyocytes were originated from BADSCs in comparison with those studies using MSCs [77], which should have contributed to the myocardial improvement. Of course, paracrine secretion should be another important contributor of BADSCs in tissue repair like MSCs. Traditionally, it was supposed that brown adipose was less distributed in adults and thus, its clinical usage in future would be limited. However, studies in recent years confirmed that brown adipose also largely existed in adult humans [83]. In addition, progress was achieved too in recent years about how to convert white adipose tissues into brown ones [84]. These reports suggested that it would be possible to obtain enough brown adipose tissues from adult human in future.

## Conclusions

In summary, this study demonstrated that SWCNT modification could significantly improve the bioactivity of heart ECM, resulting in a HH which could be used as scaffold for cardiac tissue construction and injectable carriers for stem cell delivery. It was shown that incorporated SWCNT in HH could form an integrin-dependent niche *via* the interaction with ECM proteins, which will activate integrin-related pathway and thus promote the development of primary and stem cell-derived cardiac cells towards functional tissues. When used as scaffolds for stem cell-based myocardial repair, significantly improved myocardial regeneration and repair were achieved compared with stem cell alone. Collectively, the HHs may represent a promising bioactive scaffold for regenerative medicine.

## Supplementary Information

**Additional file 1.** Additional information.

**Additional file 2.** Additional Movie 1.

**Additional file 3.** Additional Movie 2.

**Additional file 4.** Additional Movie 3.

## Data Availability

All data generated or analyzed during this study are included in this published article.
