# Correction: Conductive single-wall carbon nanotubes/extracellular matrix hybrid hydrogels promote the lineage-specific development of seeding cells for tissue repair through reconstructing an integrin-dependent niche

**DOI:** 10.1186/s12951-024-02867-w

**Published:** 2024-10-10

**Authors:** Rui Bai, Jianfeng Liu, Jiao Zhang, Jinmiao Shi, Zhigeng Jin, Yi Li, Xiaoyu Ding, Xiaoming Zhu, Chao Yuan, Bingshui Xiu, Huiliang Liu, Zengqiang Yuan, Zhiqiang Liu

**Affiliations:** 1 Senior Department of Cardiology, The Sixth Medical Center of PLA General Hospital, Beijing, 100048 China; 2 Beijing Institute of Basic Medical Sciences, Beijing, 100850 China; 3 Department of Cardiology, The Second Medical Center & National Clinical Research Center for Geriatric Diseases, Chinese PLA General Hospital, Beijing, 100853 China; 4 Department of Cardiology, Beijing Electric Power Hospital, State Grid Corporation of China, Beijing, 100073 China

**Correction: J Nanobiotechnol (2021) 19:252** 10.1186/s12951-021-00993-3

Following publication of the original article the authors identified an error in the Fig. 3.

The author found an image error in Fig. 3g (Calcium transient detection) that the two similar images from the same sample were used for HH0.5 and HH1 14 day groups due to their carelessness. The authors have carefully checked all the original data and experimental records with the first author to determine the cause of the error. The original data of Calcium transient detection was video file because calcium ion activity was dynamic. The images were taken from video file as representative results. By checking original data, they found that the two similar images (Fig. 3g in the article, HH0.5 14 day and HH1 14 day) were from two different videos, but both of them belonged to HH0.5 14 days group (Both of the video files were named as HH0.5 14 day). However, the two similar images were named as 0.5 14 d and 1.0 14 day, respectively. The authors supposed that the images were named incorrectly during image saving from videos due to carelessness of the first author, which resulted in the image error in the paper. Authors have provided corrected Fig. 3g. The authors apologize for this error.

**Uncorrected Fig. 3** 
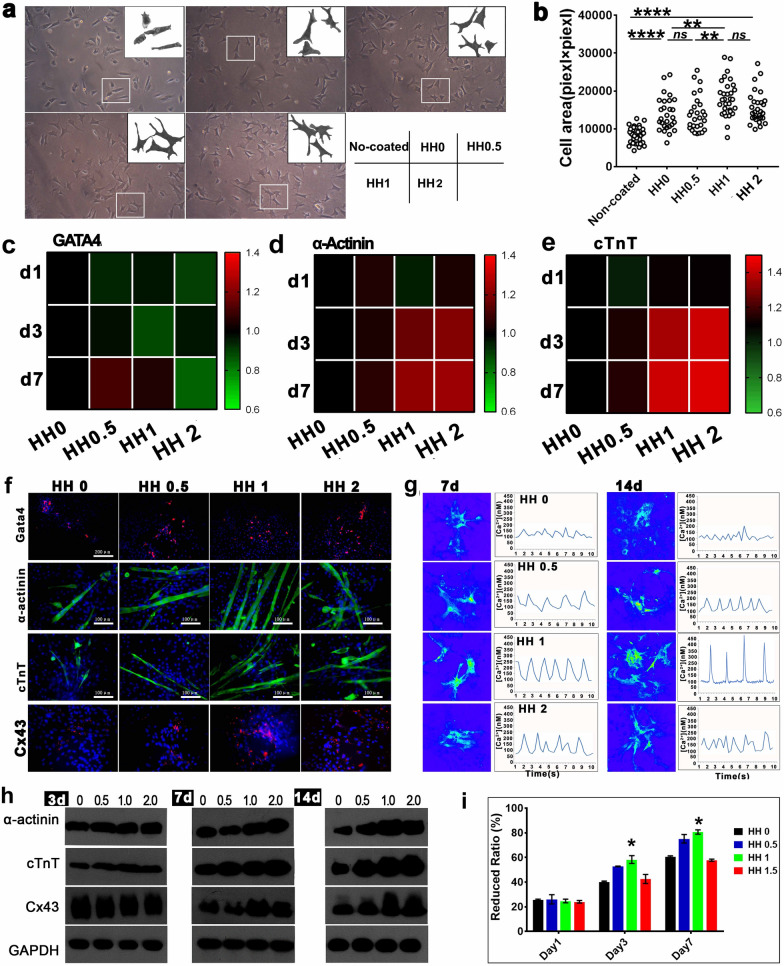


**Corrected Fig. ** **3**

**Fig. 3:**
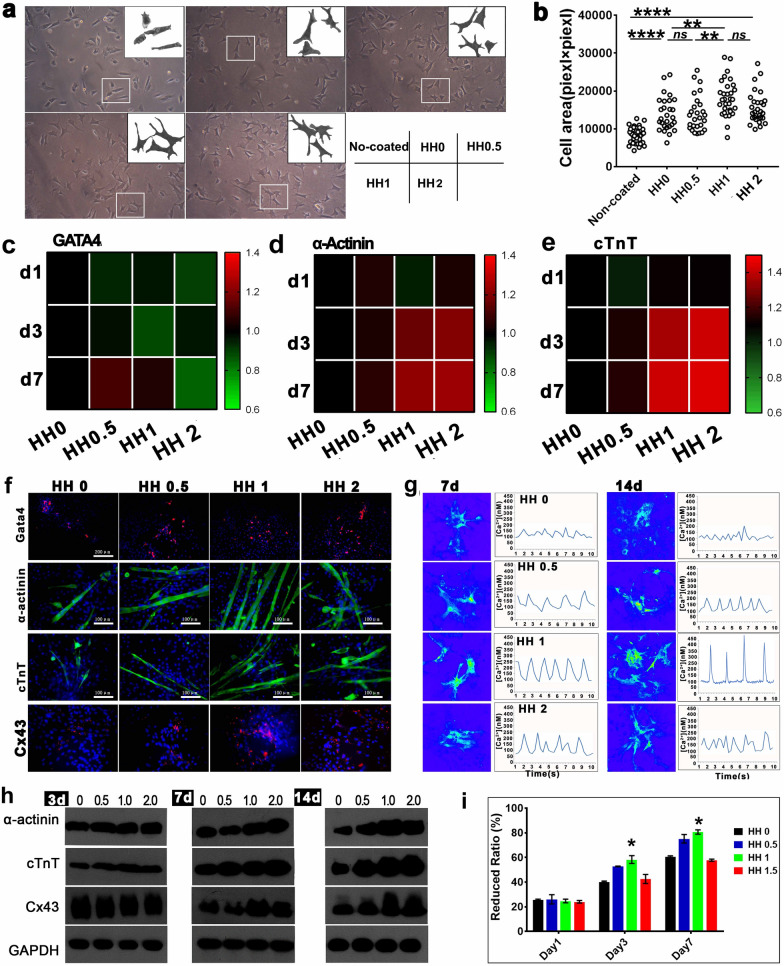
The adhesion, survival and differentiation of BADSCs on HHs. **a** The morphology of BADSCs on HHs; **b** The spreading area of BADSCs on HHs; **c**–**e** Thermography of cardiac genes in cells growing on HH-coated substrate;** f** cardiac differentiation of BADSCs on HH-coated substrate; **g** Intracellular calcium ion current of BADSC-derived cardiomyocytes growing on HH-coated substrate; **h** western blotting detecting the expression of cardiac markers in BADSCs growing on HH-coated substrate; **i** the prolifieration of BADSCs on HH-coated substrate by Alamar Blue Assays (*p < 0.05; **p < 0.01; ***p < 0.001)

The original article has been corrected.

