# Supplementary material for: Conductive single-wall carbon nanotubes/extracellular matrix hybrid hydrogels promote the lineage-specific development of seeding cells for tissue repair through reconstructing an integrin-dependent niche
Source: J Nanobiotechnology. 2021 Aug 23;19:252. doi: 10.1186/s12951-021-00993-3 (PMC8381546; doi:10.1186/s12951-021-00993-3)
Supplement: Supplementary file 1 — Additional file 1. Additional information. [file 12951_2021_993_MOESM1_ESM.doc]

**Additional file 1**

**Conductive Single-Wall Carbon Nanotubes/Extracellular Matrix Hybrid Hydrogels Promote the Lineage-Specific development of Seeding cells for Tissue Repair through Reconstructing an Integrin-Dependent Niche**

Rui Bai^1*^, Jianfeng Liu^3*^, Jiao Zhang^4^, Jinmiao Shi^2^, Zhigeng Jin^1^, Yi Li^1^, Xiaoyu Ding^2^, Xiaoming Zhu^2^, Chao Yuan^2^, Bingshui Xiu^2^, Huiliang Liu^1#^, Zengqiang Yuan^2#^, Zhiqiang Liu^2#^

1. Senior Department of Cardiology, the Sixth Medical Center of PLA General Hospital, Beijing, 100048, China.

2. Beijing institute of basic medical sciences, Beijing 100850, China

3. Department of Cardiology, the Second Medical Center & National Clinical Research Center for Geriatric Diseases, Chinese PLA General Hospital，Beijing，100853, China.

4. Department of Cardiology, Beijing Electric Power Hospital, State Grid Corporation of China, Beijing, 100073, China.

* The authors contributed equally to this work.

# Correspondance to Huiliang Liu (lhl518@vip.sina.com), Zengqiang Yuan (zyuan620@yahoo.com) and Zhiqiang Liu (zhiqiangliu_amms@163.com)

**Materials and Methods**

**Preparation and characterization of SWCNT-ECM hybrid hydrogels**

Adult Sprague-Dawley rats (SD, ~250g) were used as heart donors. Animals were anesthetized by [intraperitoneal injection](http://www.baidu.com/link?url=iTBj_nDD8FOHVQxj1DnXOkGIQ3aed1Jjkg-KgccXWLpft5SczbxvGc8GKtbGXeBWuPvMGeiD7TS6SUsqjjJO4GRe6a7WoJEWZnOmyV4sWytkzpr-uU3lNQn91Sib6nLb) of pentobarbital natricum (30mg/Kg body weight) and then, thoracotomy was performed. 10 IU/ml heparinized PBS was injected through inferior vena cava to prevent blood coagulation. Hearts were explanted and bloodiness was removed by extensively washing with sterile PBS. A 16G blunt end needle was inserted into the aorta for retrograde perfusion. Hearts were perfused with 1% SDS for 6 hours followed by 1% Triton X-100 for 0.5h. After washing with PBS, decellularized hearts were lyophilized to obtain solid ECM. Pepsin-HCl solution was used to digest the solid ECM for preparing ECM solution. For gelation, NaOH was used to neutralize excess acid. SWCNT-ECM hybrid hydrogels (HHs) were prepared by adding single wall carbon nanotube (SWCNT, US nanomaterials research) at different final concentration.

For transmission electronic microscopy (TEM), HH was dropped on a special copper net. After air dry naturally, TEM was performed. For scanning electron microscopy (SEM), HH was lyophilized and prepared as 0.5 cm×0.5 cm blocks. After fixation onto sample table with conducting adhesive, they were placed in Gatan Precision Etching Coating System (PECS) for carbon spraying treatment. The conductivity of materials was tested by electrochemical workstation. With 1V measurement voltage, the current through HH was detected at the interval of 0.1s. 4 samples were detected for each material. The electrical resistance was calculated as voltage/current. The conductivity was expressed as 1/electric resistance. Young’s modulus measurement was performed according to the previous report .

**Cultivation and characterization of cardiac cells**

Cardiomyocytes were isolated from neonatal rats according to the previous report and cultured with DMEM supplemented with 10% FBS (fetal bovine serum, Gibco). To determine the influence of HH on the adhesion and survival of cardiomyocytes, HH with different SWCNT concentration (0, 0.5, 1 and 2 mg/mL) were used to coat the plate for 1h before cell seeding. 2×10^4^ cells were seeded onto 24-well plate in DMEM/10% FBS and incubated for 6h in 37℃, 5% CO_2_ environment. Cell adhesion was assessed by counting cell numbers per field under microscope. Cell spreading area was analyzed using Image Pro Plus software and expressed as pixel×pixel. The expression of integrin β1 was determined by immunostaining and western blotting respectively as described in the following sections. To evaluate the cell viability, Live/Dead staining was performed using a commercial kit (Invitrogen) according to manufacturer’s guidance.

**Real time PCR and Western Blotting**

Quantative real-time PCR was performed to detect the gene expression. The gene-specific primers were as follows: 5’-GGAAGACTGGAGCGAAGA-3’ (Forward) and AAGTTGGGCATGAAGAGC (Reverse) for cTnT; 5’-AGCCCACGACCAGTTCA-3’ (Forward) and 5’-CTTCCAGAGTCCCGTTCA-3’ (Reverse) for α-Actinin; 5’-AAGAAGCCTGCATTCTCACA-3’ (Forward) and 5’-AAGCCTCGGTCCCTACA-3’ (Reverse) for Gata4; 5’-GCAAGTTCAACGGCACAG-3’ (Forward) and 5’-GCCAGTAGACTCCACGACAT-3’ (Reverse) for GAPDH. The gene expression was normalized with GAPDH and quantified using the 2^-ΔΔCt^ method.

Cells were collected and lysed using Laemmli Sample Buffer (Bio-Rad) for protein extraction. The proteins were quantified with BCA protein assay kit (Pierce, Thermo Scientific, USA). Protein electrophoresis was performed with 10 % SDS-containing polyacrylamide (SDS-PAGE) gel. For each sample, a total of 40ug protein was loaded. To separate protein bands, electrophoresis was performed at 80V voltage for 20min and then at 120V until the end. The separated proteins in gel were then electronically transferred onto PVDF membrane (Roche) in ice bath. Before incubation with primary antibodies, the PVDF membrane was blocked with 5% non-fat milk for 1h at room temperature. Primary antibodies, including cTnt (1: 1000, abcam), Cx 43(1: 2000, abcam), α-Actinin(1:2000, abcam) and GADPH (1: 5000, abcam), were incubated with membrane overnight at 4℃. After washing with TBST for three times, HRP conjugated secondary antibodies (Beyotime, Beijing) were added and incubated with membrane for 2h at room temperature. Chemiluminiscence was performed to detect protein band using Clarity™ ECL Western Blotting Substrate kit (Bio-Rad Laboratories, Inc., USA). Then, a photographic film was used to visualize protein bands.

**Preparation of 2D and 3D cardiac tissues**

To prepare 2D cell sheet, 1cm × 1cm sized glass slides were used and coated with HH. Glass slides were firstly treated with concentrated sulfuric acid followed by extensive washing with sterile water. Then, the slides were immersed in ethanol for 1-2h. After air dry naturally, 150μL HH solution was added onto slides evenly. The slides were dried at 37℃ and a thin layer of HH substrate was formed on the slide. Before cell seeding, the slides were placed into a 24-well plate, 5×10^4^ cells were seeded onto one slide and cultured at 37℃, 5%CO_2_ incubator.

To construct 3D cardiac tissue, the previously reported method was referenced. Briefly, 2% agarose solution was added into 24 or 48-well plates (300μL/well for 48 well plates and 500μL/well for 24 well plates). After solidification, capillary glass tubes of suitable length were inserted into agarose gel for fixing cardiac tissues during culture. HH solution was mixed with 2×α-MEM at the ratio of 1:1. Then, BADSCs were suspended in HH solution at a concentration of 2.5×10^6^/mL and pH was adjusted to be neutral. The cell suspension was poured into 24 (200μL/well) or 48-well plates (100μL/well). The plates were then placed into 37℃, 5% CO_2_ environment for gelation. α-MEM supplemented with 10% FBS was used for cultivation.

**PET/CT imaging**

PET/CT imaging was performed using a Super Nova PET/CT（SNPC-103）instrument (PINGSENG Healthcare Inc) according to the previous report . Briefly, four weeks after cell transplantation, ^18^F-FDG was injected into each rat through tail vein as the previous report . After 40min metabolism, rats were anesthetized by inhalation of 2% isoflurane and imaging data was acquired.

**Echocardiogram**

Cardiac function was assessed through echocardiogram at 4week after surgery. Briefly, rats were anesthetized by intraperitoneal injection of pentobarbital sodium. An echocardiography (14.0 MHz, Sequoia 512; Acuson) was applied and several functional parameters were measured by a skilled technician who was blind to the study, including left-ventricular end-diastolic diameter (LVEDD), left-ventricular end-systolic diameter (LVESD), left ventricular shortening fraction (LVFS) and left ventricular ejection fraction (LVEF).

**Histology**

For cell tracking, some animals were euthanized with overdose of sodium pentobarbital at 1 week after transplantation. Hearts were explanted. Frozen sections were prepared and nuclei were stained with DAPI. DiI positive cells were detected under laser confocal fluorescence microscope. At 4week after surgery, animals were euthanized with the same method above. Hearts were fixed with 4% paraformaldehyde and parafﬁn-embedded sections were prepared; Masson’s Trichrome and H&E staining were performed according to the standard procedure. Infarct size, left heart wall thickness and fibrosis of infarct area were analyzed using Image Pro Plus software.

**Immunohistochemistry**

Immunostaining was performed according to the standard protocol. Briefly, cells or tissue sections were firstly permeabilized with 0.3% Triton X-100 in PBS for 30 min and then, incubated with 2% BSA solution for 1h. Primary antibodies were incubated with samples overnight at 4℃ according to manufacturers’ instructions. After washing with PBS, fluorescein or HRP-conjugated secondary antibodies were added and incubated at room temperature for 2h. Nuclei were stained with DAPI or hematoxylin. HRP-labeled antibodies were further visualized with DAB kit (Beyotime, Beijing) according to manufacturer’s instruction. Antibodies for connexin 43, α-actinin, gata4, cTnT and p-Akt were purchased from Abcam; antibodies for integrin β1, p-FAK and paxillin were purchased from Santa cruz biotechnology. Anti-GAPDH and anti-VWF antibodies were purchased from Beyotime.


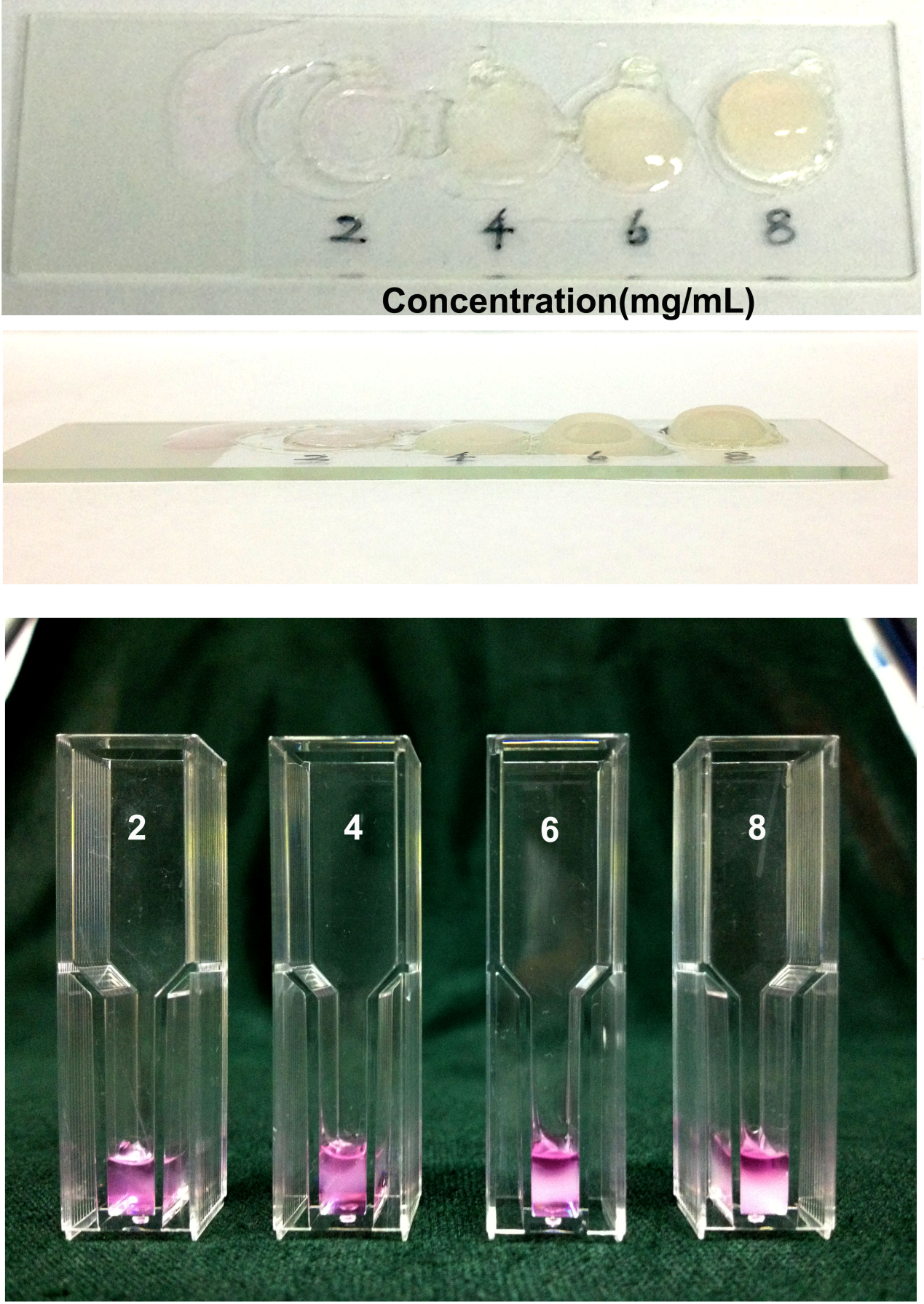


**Figure S1 Gelation of decellularized heart ECM hydrogel at different concentration.**

**
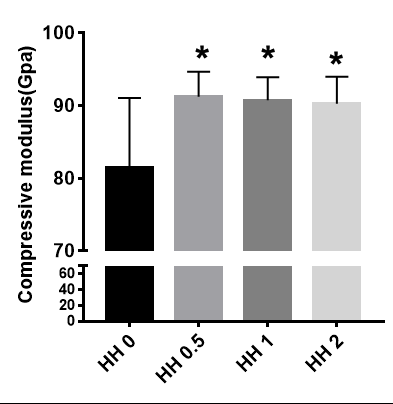
**

**Figure S2 Compression modulus of HH containing different dose of SWCNT.** HH 0: HH containing no SWCNT (Pure ECM hydrogel); HH 0.5: HH containing 0.5mg/mL SWCNT; HH 1: HH containing 1mg/mL SWCNT; HH 2: HH containing 2mg/mL SWCNT(* P<0.01 compared with HH0).


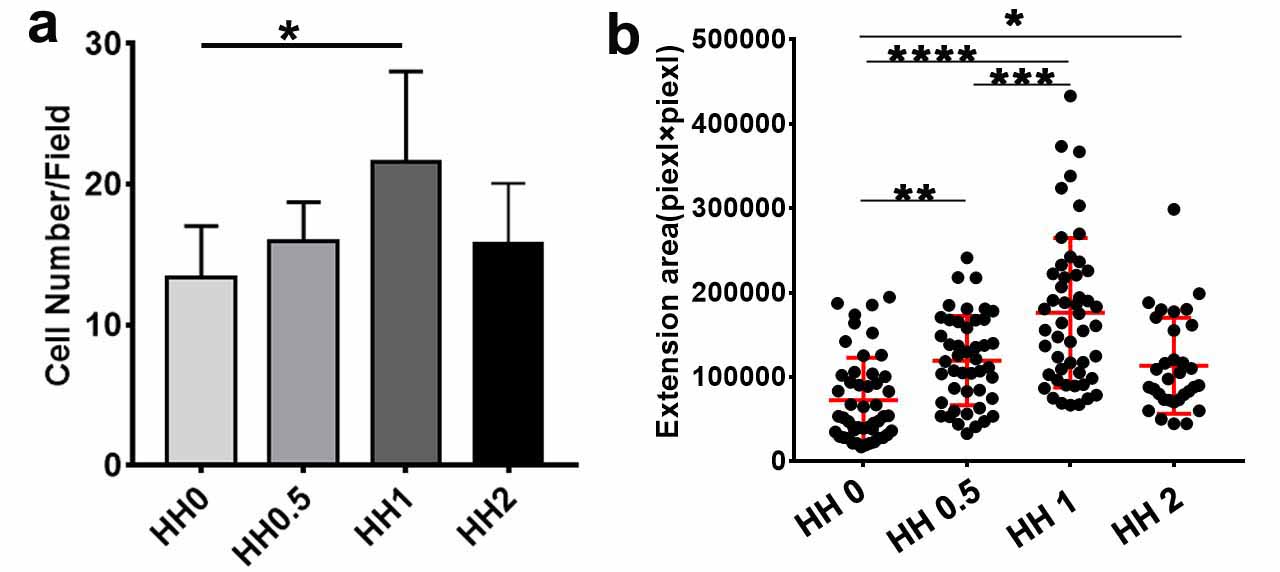


**Figure S3 The adhesion and spreading of cardiac cells on HH.** a**,** Comparison of adhered cells on HH-coated matrix; b, comparison of the cell spreading growing on HH-coated substrate **(***P<0.05; **P<0.01; ***P<0.001; ****P<0.0001).


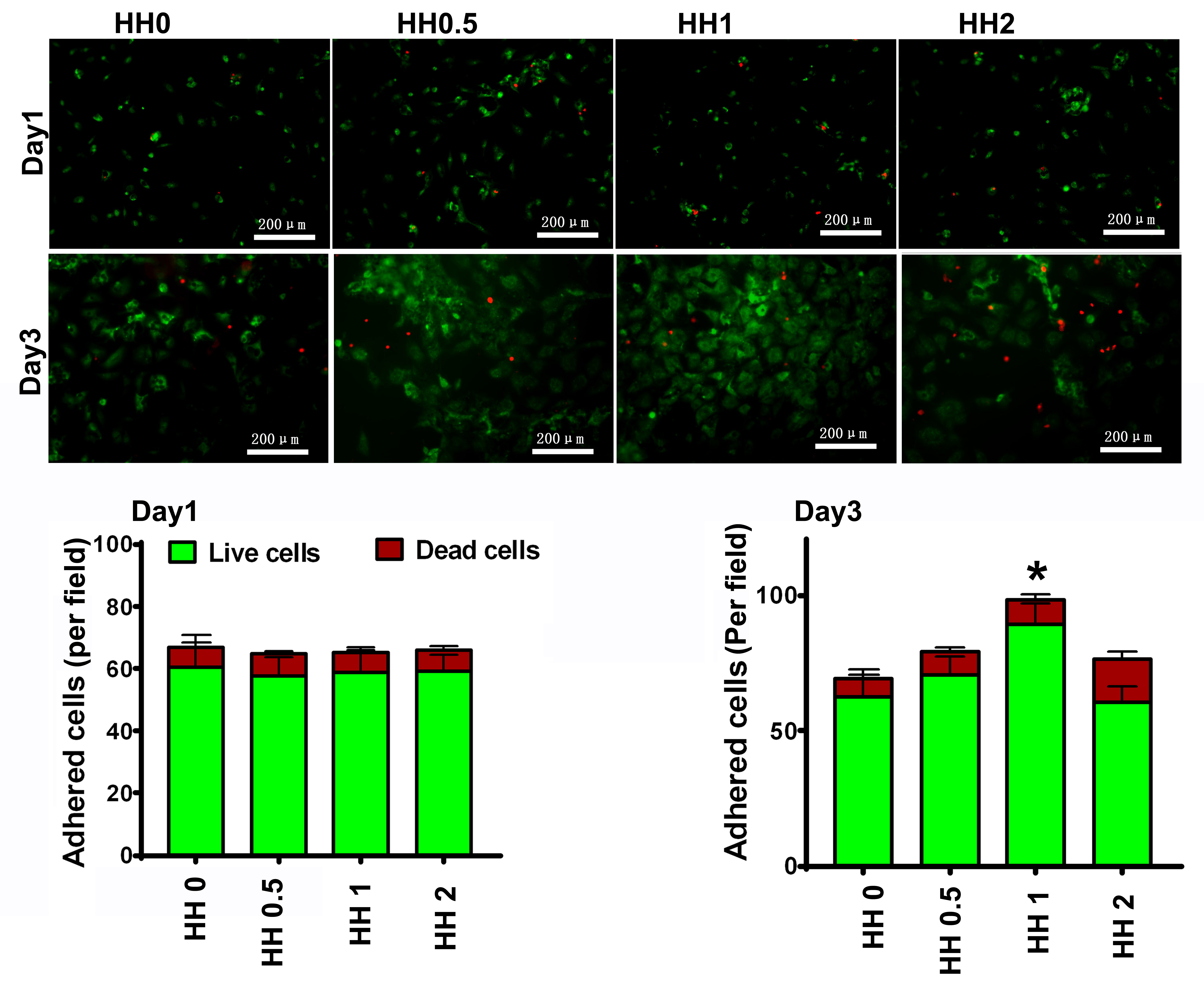


**Figure S4 Adhesion and survival of primary cardiac cells on HH-coated plates.** **P*<0.01 compared with HH 0.


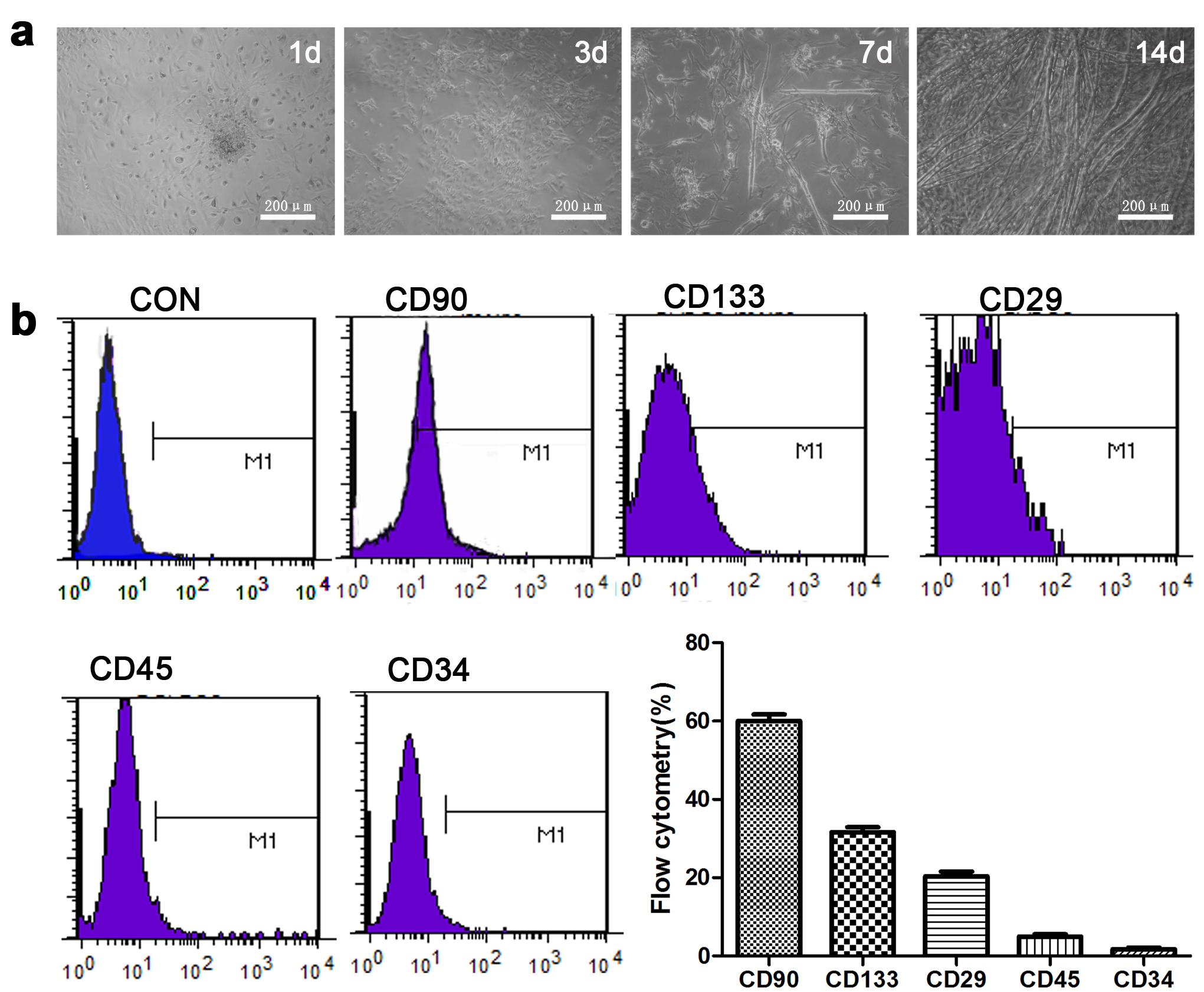


**Figure S5 isolation and characterization of BADSCs.** a, the morphology of ultured BADSCs; b, Flowcytometry analysis of surface markers of BADSCs.


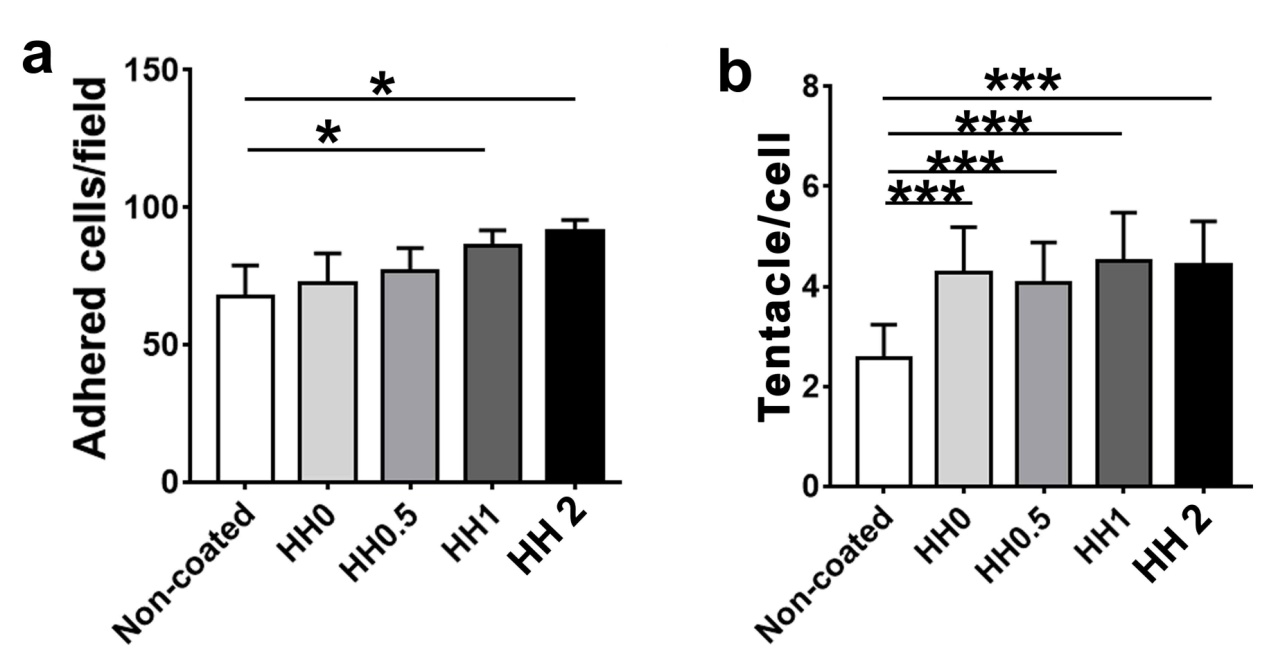


**Figure S6 Adhesion and tentacle outgrowth of BADSCs on different matrix.** a, comparison of adhered cells on HH-coated matrix; b, comparison of the tentacle outgrowth from cells growing on HH-coated substrate. (**p*<0.05; ***p*<0.01; ****p*<0.001)


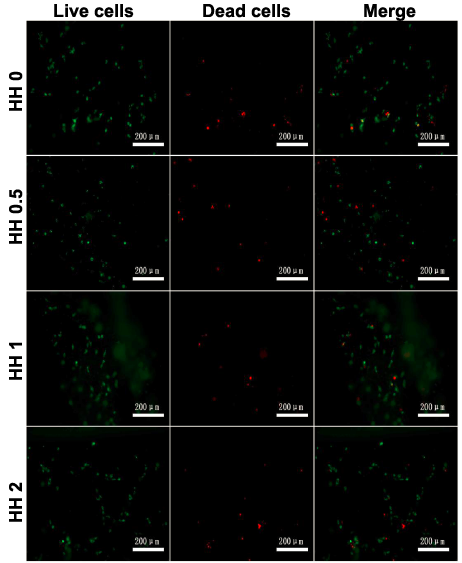


**Figure S7 Live/Dead staining of BADSCs growing on HH-coated plates for 1day.**


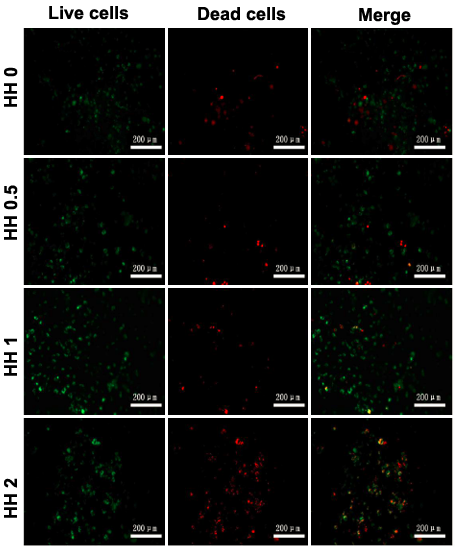


**Figure S8 Live/Dead staining of BADSCs growing on HH-coated plates for 3day.**

**
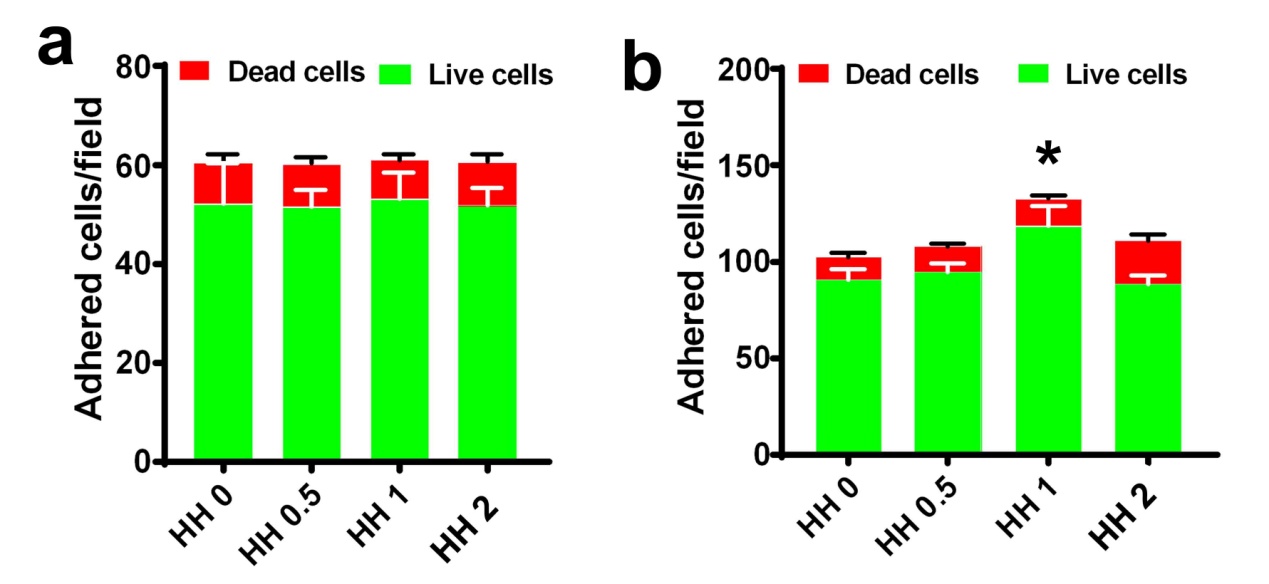
Figure S9. Cell viabilities during growth on different matrix.** a, cell viabilities of BADSCs after one day’s culture on HH-coated substrate; b, cell viabilities of BADSCs after three day’s culture on HH-coated substrate;


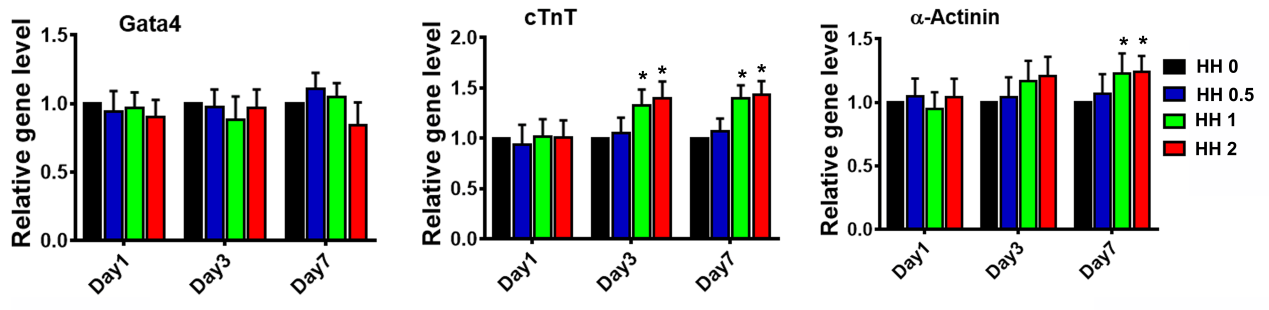


**Figure S10. RT-PCR detecting the expression of cardiac genes in BADSCs growing on HH-coated plates.** **P*<0.05 compared with HH 0.


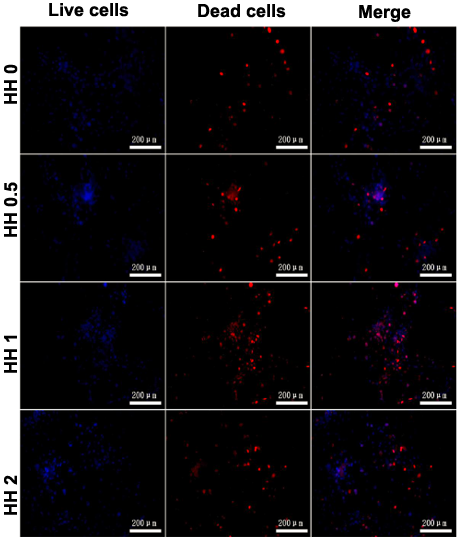


**Figure S11. BrdU staining of BADSCs growing on HH-coated plates.**

**
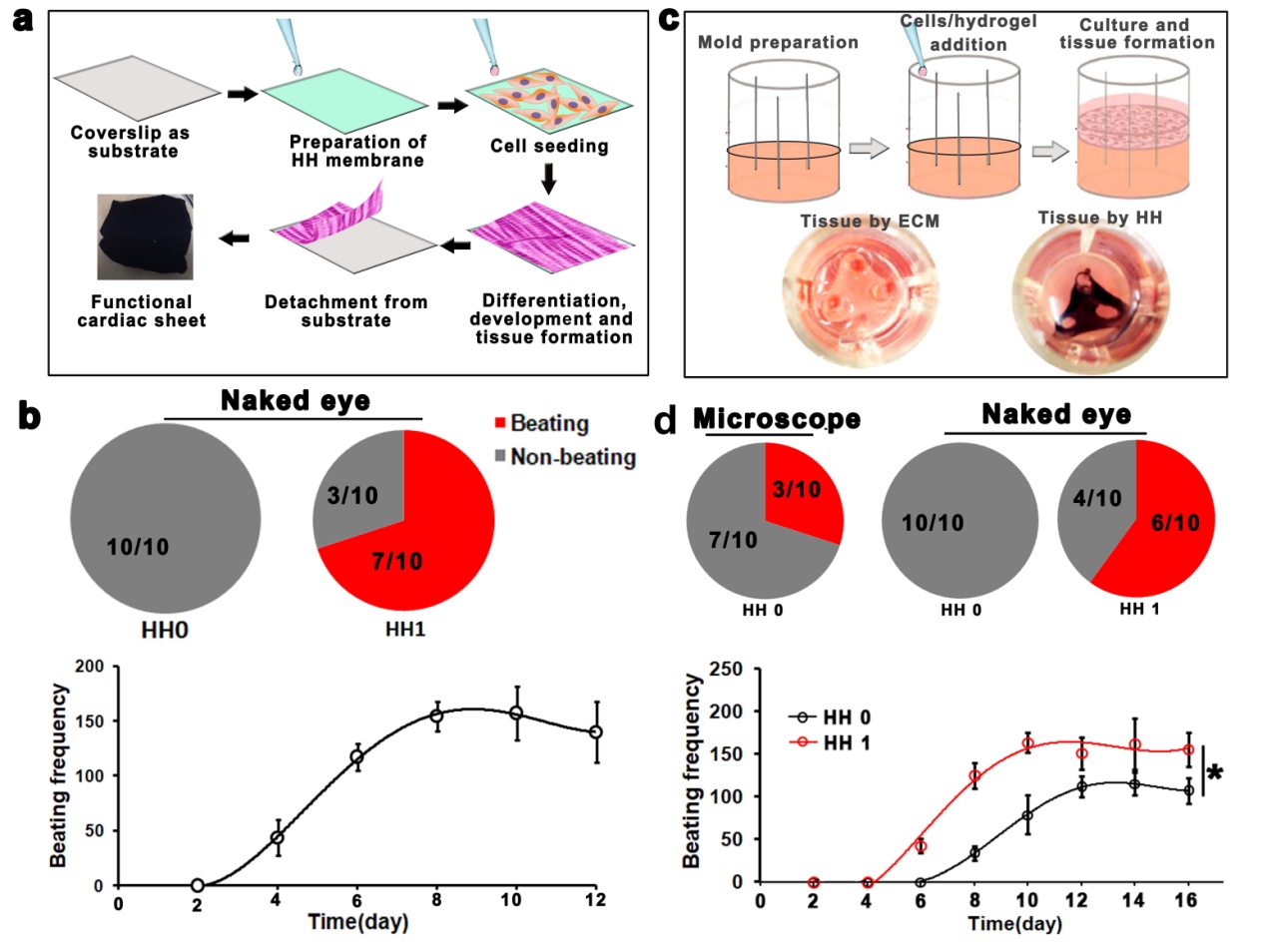
**

**Figure S12. Construction of 2D and 3D engineered cardiac tissues.** a, schematic illustrating the preparation of 2D cardiac sheets using BADSCs; b, formation of rhythmic beating sheets from BADSCs growing on 2D HH film; c, schematic illustrating the preparation of 3D cardiac stissues using BADSCs; d, formation of 3D rhythmic beating tissues from BADSCs growing in HH.

**
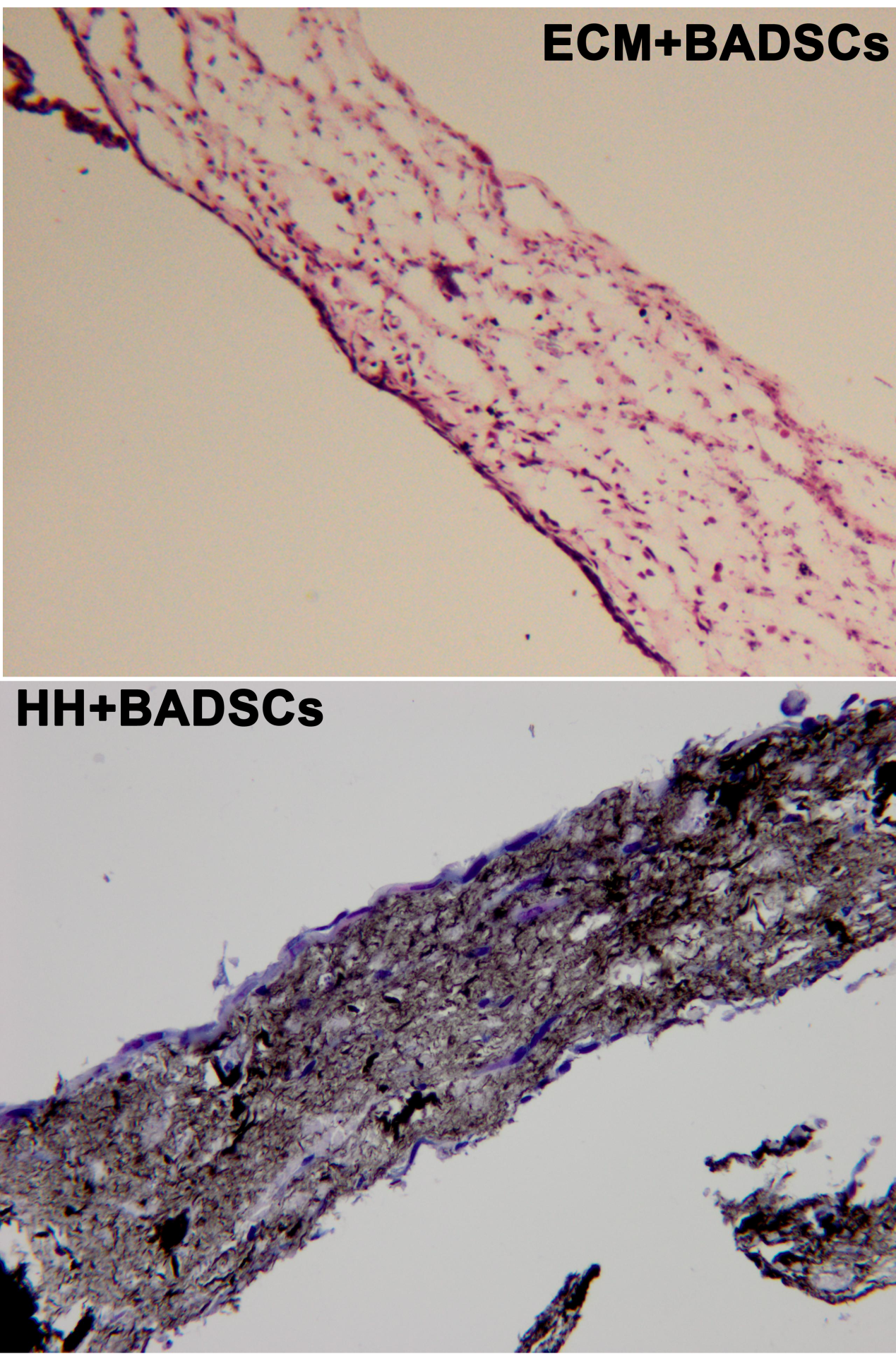
**

**Figure S13. The H&E staining of 3D cardiac tissues.**

**
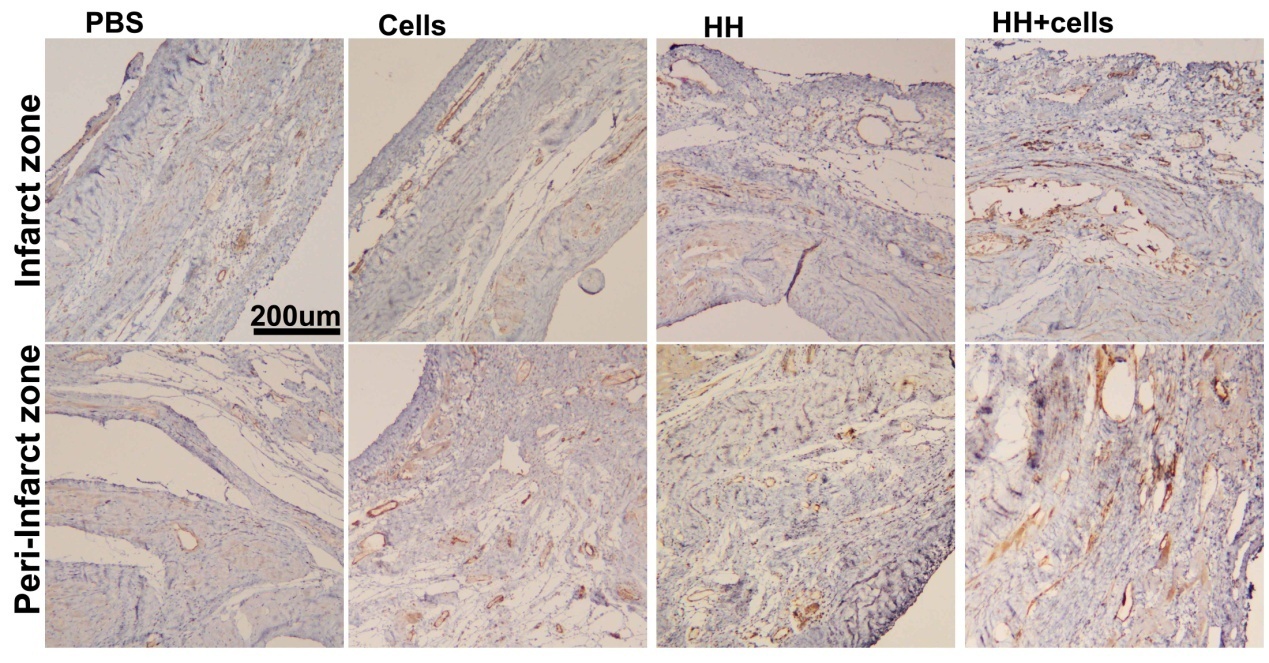
**

**Figure S14. Anti-vWF staining on heart sections from different groups.**

**

**

**Figure S15 Immunostaining against α-actinin on heart sections 4w after BADSC transplantation**

**Reference**

[1] Li Y, Shi X, Tian L, Sun H, Wu Y, Li X, et al. AuNP-Collagen Matrix with Localized Stiffness for Cardiac-Tissue Engineering: Enhancing the Assembly of Intercalated Discs by β1-Integrin-Mediated Signaling. Advanced materials (Deerfield Beach, Fla). 2016;28:10230-5.

[2] Liu Z, Wang H, Wang Y, Lin Q, Yao A, Cao F, et al. The influence of chitosan hydrogel on stem cell engraftment, survival and homing in the ischemic myocardial microenvironment. Biomaterials. 2012;33:3093-106.

[3] Lü S, Li Y, Gao S, Liu S, Wang H, He W, et al. Engineered heart tissue graft derived from somatic cell nuclear transferred embryonic stem cells improve myocardial performance in infarcted rat heart. Journal of cellular and molecular medicine. 2010;14:2771-9.

[4] Quintana-Villamandos B, Delgado-Martos MJ, Fernandez-Riveira C, Fernández-Criado MC, Martos-Rodríguez A, Canillas F, et al. Can 18F-FDG-PET show differences in myocardial metabolism between Wistar Kyoto rats and spontaneously hypertensive rats? Laboratory animals. 2013;47:320-3.

[5] Radford KD, Park TY, Jaiswal S, Pan H, Knutsen A, Zhang M, et al. Enhanced fear memories and brain glucose metabolism ((18)F-FDG-PET) following sub-anesthetic intravenous ketamine infusion in Sprague-Dawley rats. Translational psychiatry. 2018;8:263.
